# Supplementary material for: Nonlinear optomechanical measurement of mechanical motion
Source: Nat Commun. 2016 Mar 21;7:10988. doi: 10.1038/ncomms10988 (PMC4802172; doi:10.1038/ncomms10988)
Supplement: Supplementary Information — Supplementary Figures 1-8, Supplementary Table 1, Supplementary Notes 1-11 and Supplementary References [file ncomms10988-s1.pdf]

SUPPLEMENTARY INFORMATION
Non-linear Optomechanical Measurement of Mechanical Motion

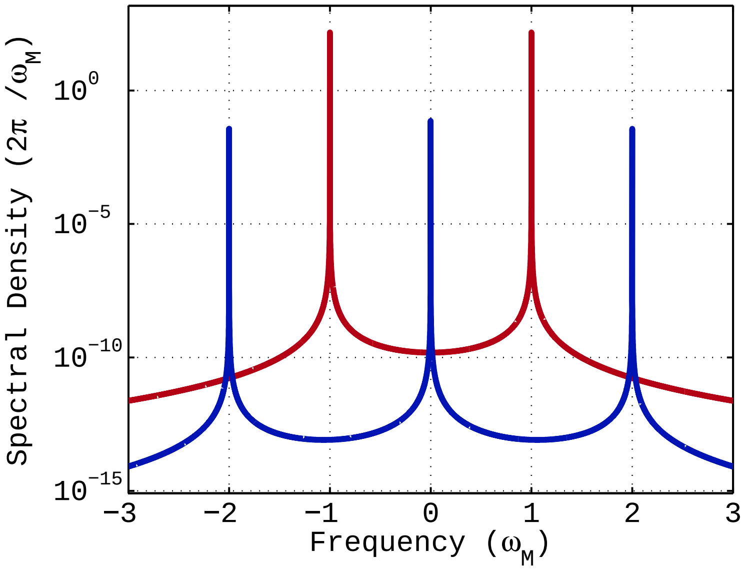

Supplementary Figure 1: Plot showing the mod-square of the mechanical response function (red) and the convolution of this function with itself (blue). Normalisation is identiacal to that used in Eq. (30) and Eq. (39) respectively, with $\bar{n} = 6 \times 10^7$ and $\omega_{\rm M}/\gamma = 10^6$.

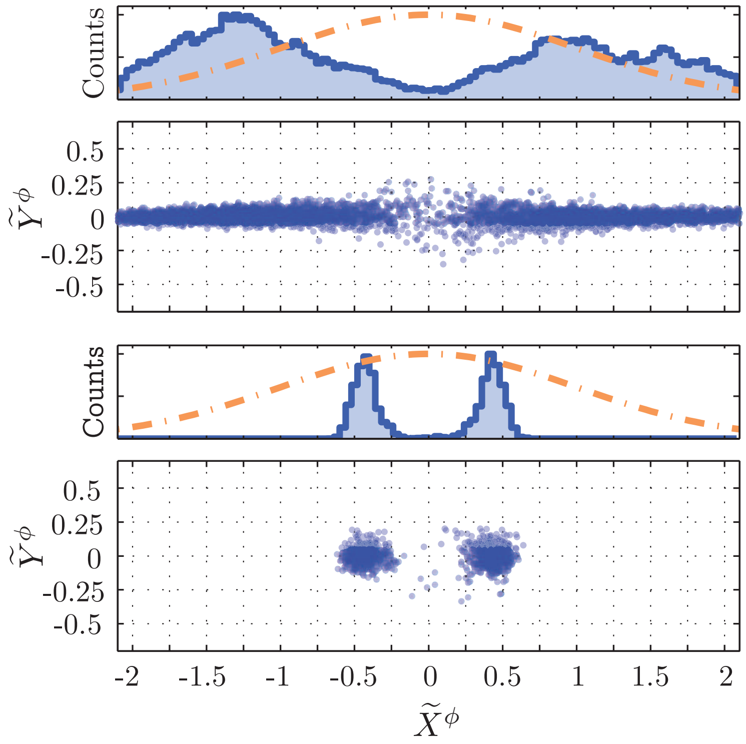

Supplementary Figure 2: In the upper plot, the linear acausal data $(\tilde{X}; \tilde{Y})$ is conditionally rotated according to transformation (58), conditioned on the causal quadratic data $(\tilde{P}; \tilde{Q})$. In the lower plot, the additional restriction of $2|c| = 0.2$ is made, localising the state into two small regions at $\pm\sqrt{0.2}$.

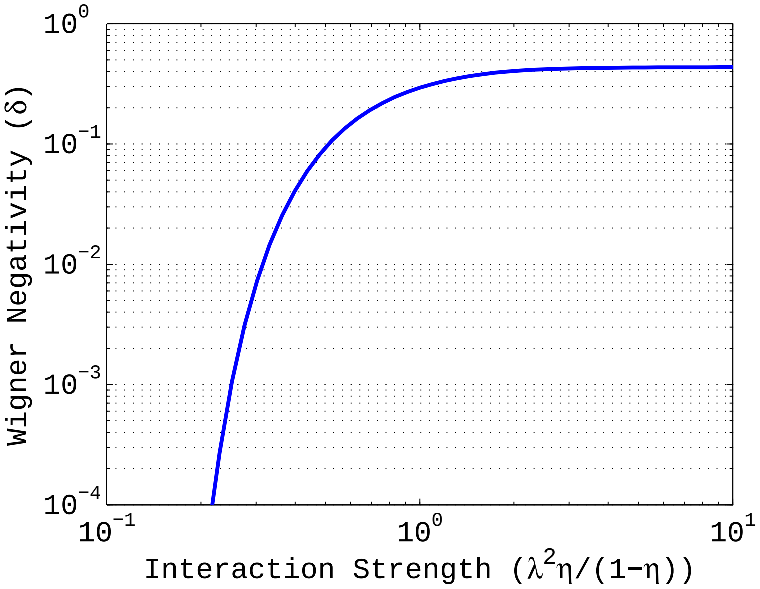

Supplementary Figure 3: Here the quadratic interaction $\eta\chi_2^2$ is set constant and the coupling parameter λ is varied, which is equivalent to changing the strength of the linear interaction. For small λ, moderate optical loss suppresses the appearance of negativity, whilst for larger values of λ, the negativity eventually saturates at a value given by a pure X^2 measurement. For comparison, an even (pure) coherent state superpostion with the same separation has a computed negativity of $\delta \approx 0.6$.

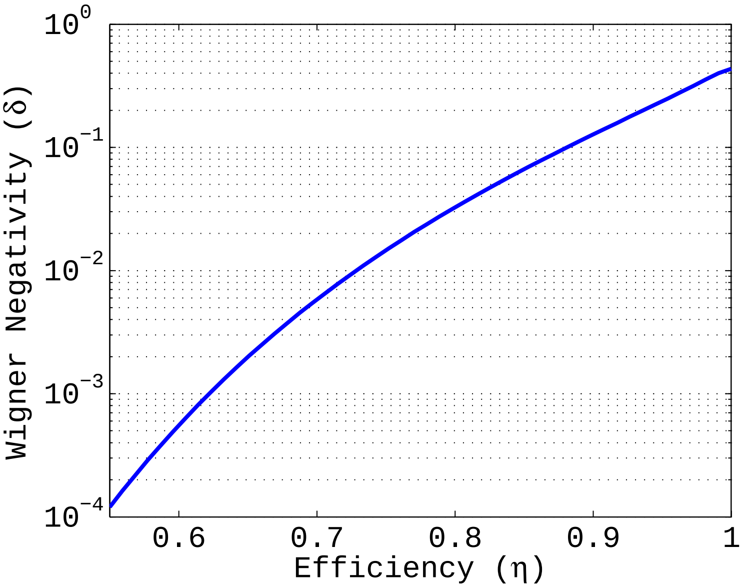

Supplementary Figure 4: The maximum Wigner negativity attained for a separation of $X^2_{\rm M,est} = 3^2$ at a range of different efficiencies. Here the initial state is the mechanical ground state, and the interaction strength chosen correspond to an intracavity photon number of $N = 30$ and coupling strength of $\lambda = 0.2$. Plots of the Wigner functions of these states are shown in Fig. 5.

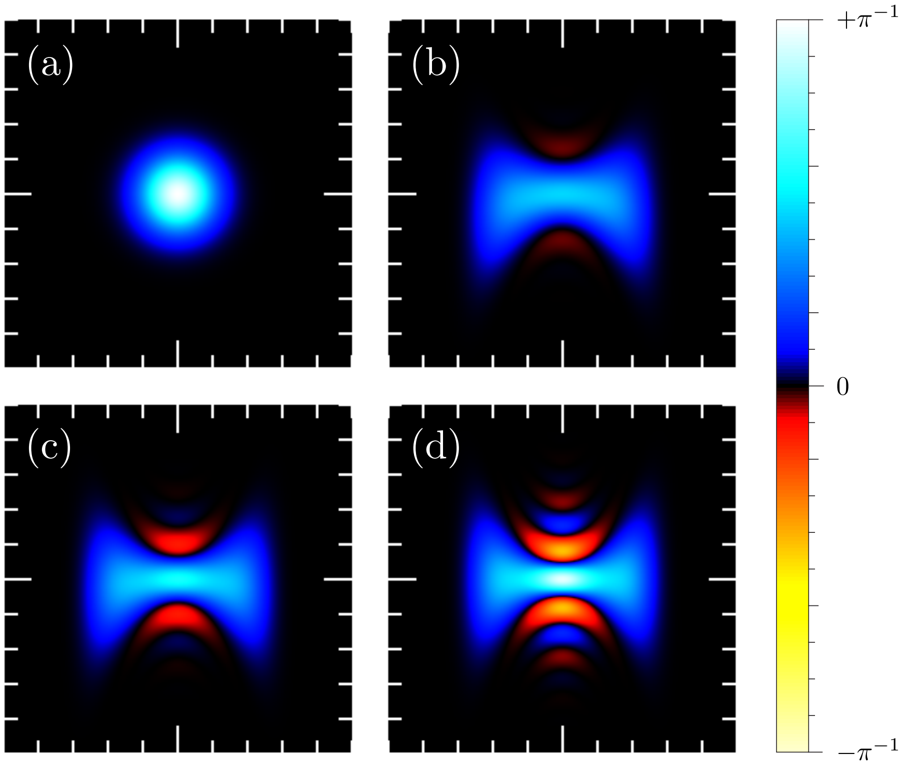

Supplementary Figure 5: **Simulated Wigner functions of the mechanical state, conditioned on a fixed quadratic measurement outcome.** **(a)** The initial mechanical ground state. **(b-c)** Conditional state with detection efficiencies: $\eta = [0.8,\ 0.9,\ 0.99]$; $N_C = 30$ and $\lambda = 0.2$. Note that the separation of the peaks in the position marginal only approaches the conditioning value in the limit of arbitrarily strong quadratic measurement strength (see [5] for further details).

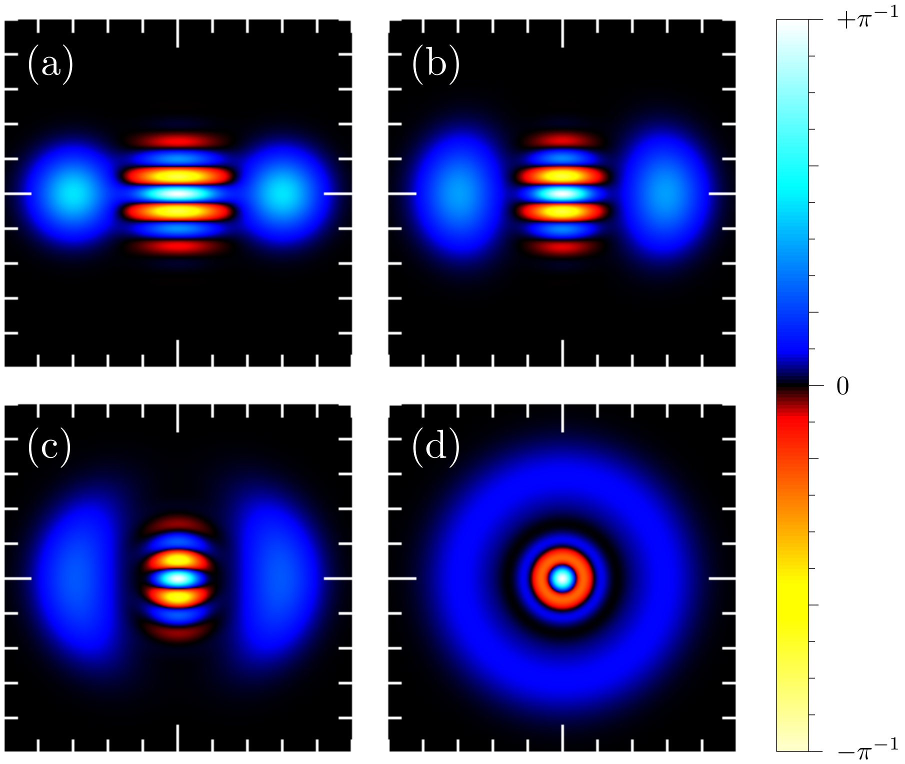

Supplementary Figure 6: **Simulated Wigner functions of a mechanical oscillator in a superposition of coherent states undergoing decoherence via the environment coupling to the phonon number. (a)** The initial mechanical state $|\psi\rangle \propto |3/\sqrt{2}\rangle + |-3/\sqrt{2}\rangle$. **(b)** A small amount of decoherence ($\theta = 0.3\sqrt{2}$). **(c)** A modest amount of decoherence ($\theta = 0.6\sqrt{2}$) **(d)** A very large amount of decoherence ($\theta \to \infty$). Note that non-classicality remains even as the environment gains complete knowledge of the phonon number.

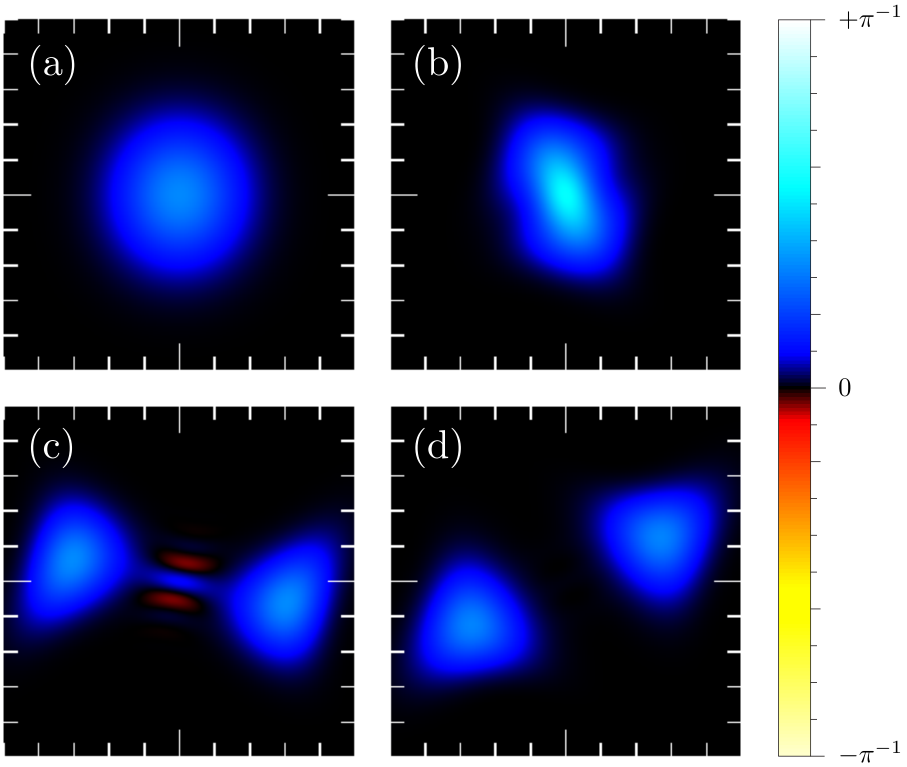

Supplementary Figure 7: Typical trajectory of the Stochastic Master Equation Simulation of continuous measurement of the mechanics, including number decoherence and finite detection efficiency. Figure **(a)** shows the initial thermal state with $\bar{n} = 1$ inital purity $\gamma = 0.33$ and negativity $\delta = 0$; **(b)** at time $t = 4.1T$, that action of the nonlinear measurement has increased the state purity to $\gamma = 0.52$, however the state is not yet sufficiently excited to produce a bimodal distribution; **(c)** at time $t = 6.4T$, the negativity has peaked at $\delta = 2 \times 10^{-2}$, however the purity is decreasing to $\gamma = 0.34$ due to the cats state's extreme sensitivity to environmental decoherence; **(d)** at time $t = 8.8T$, the purity has fallen to $\gamma = 0.32$ and negativity is almost imperceptible at $\delta = 1 \times 10^{-4}$.

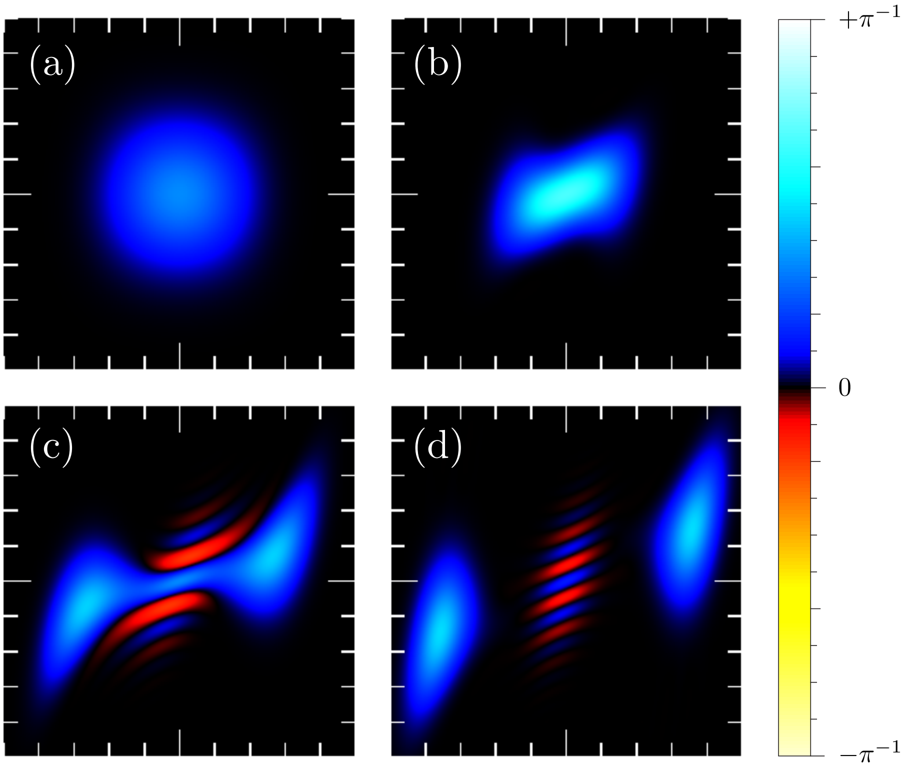

Supplementary Figure 8: **Typical trajectory of the Stochastic Master Equation Simulation of continuous measurement of the mechanics, with perfect detection efficiency and number decoherence.** Here the same coupling parameter λ and intracavity photon number N was used as before, however the detection efficiency has increased to unity, resulting in no linear decoherence from the measurement (bath decoherence and phonon number decoherence remain). Figure **(a)** shows the initial $\bar{n} = 1$ state; **(b)** at time $t = 1.2T$, $\delta = 3 \times 10^{-4}$, $\gamma = 0.72$; **(c)** at time $t = 4.6T$, $\delta = 0.18$, $\gamma = 0.54$; **(d)** at time $t = 5.0T$, $\delta = 0.090$, $\gamma = 0.50$. Note the onset of significant Wigner negativity occurs more rapidly in this case due to the more precise cancellation of radiation pressure noise.

Name	Parameter	Current Work	Electromechanical System		Cold Atoms
Temperature	T	300K	20mK	20mK	1.5μK
Mechanical Frequency	$\omega_{\mathrm{M}}/2\pi$	0.1MHz	65MHz	65MHz	0.037MHz
Interaction Strength	λ	2.9×10^{-6}	0.057	0.6	3.2
Detection Efficiency	η	10%	99.7%	75%	10%
Decoherence Process	Measurement Parameter				
Thermal Decoherence	$\eta\kappa N\lambda^4/\Gamma(\bar{n}+\frac{1}{2})$	4.7×10^{-5}	$3.1 \times 10^{2(*)}$	$3.2 \times 10^{5(*)}$	$1.8 \times 10^{6(*)}$
Detection Efficiency	$\eta\lambda^2/(1-\eta)$	1.9×10^{-12}	$1.1^{(*)}$	$1.1^{(*)}$	$1.1^{(*)}$
Lock Precision	$\eta\lambda^2/\left\langle\xi^2\right\rangle$	1.7×10^{-6}	$3.2 \times 10^{3(*)}$	$2.7 \times 10^{5(*)}$	$1.1 \times 10^{6(*)}$
Mode Mixing (uniform coupling)	$19.3\ \eta/(\bar{n}+\frac{1}{2})$	N/A	$2.8^{(*)}$	$2.1^{(*)}$	N/A
Mode Mixing (delta coupling)	(Unconditionally $= 0$)	0	N/A	N/A	N/A

Supplementary Table 1: **Decoherence parameters expressed as a ratio of the quadratic measurement rate to the effective linear decoherence rate.** For each process, a measurement parameter > 1 (highlighted with $*$) is a requirement to enter a quantum regime. Although the current proof of principle work is definitively not in a quantum regime, both the electromechanical and cold atom systems presented can reach a regime where the quadratic measurement rate dominates all decoherence rates. The first *Electromechanical System* column is reproduced from reference [10], with a speculative high detection efficiency; the second column is the same as the first, however with a single photon coupling rate that is 10 times larger, and a detection efficiency possible with state-of the art amplifiers [11]. Column *Cold Atoms* is a cold trapped atom system [12], with an assumed modest detection efficiency of 10%. All three additional systems presented are in a regime of quantum state preparation.

Supplementary Note 1: A brief introduction to measurement based state preparation

In both quantum mechanics and classical statistical mechanics the state of a system, i.e. an observer's knowledge of the system variables, can be changed by measurement as knowledge of the system changes. Here we give a brief introduction to this concept of 'conditonal' or 'measurement-based' state preparation for both classical and quantum systems. For a more detailed introduction we suggest the reader refer to Ref. [1].

Classical systems: To describe classical state preparation by measurement we will consider a measurement of a single system variable X on a system with initial probability distribution $\Pr(X)$. Typically we have some knowledge of the system prior to measurement, such as knowing the system is in thermal equilibrium at a certain temperature, which determines the form of the initial distribution. If we perform a perfect measurement of X then immediately after the measurement $\Pr(X)$ will be a delta-function centred about the measurement outcome. In practice, there will always be noise associated with a measurement and so the probability distribution conditioned on the measurement outcome will always have a finite width. It is useful to think of correlating the system of interest with a fluctuating ancillary meter to model such noise. By making an ideal measurement on the meter we can then infer, to within a certain precision, the value of X. The probability distribution of X conditioned on the measurement with outcome Q, i.e. the conditional probability distribution, is then determined by Baye's theorem

$$\Pr(X|Q) = \frac{\Pr(Q|X)\Pr(X)}{\Pr(Q)}, \tag{1}$$

where $\Pr(Q|X)$ describes the distribution of the noise on the meter for a given value of the system variable X, and $\Pr(Q)$ is the probability of obtaining outcome Q. We would like to note that this description of classical measurement has assumed that the measurement is non-disturbing or back-action evading, i.e. it does not perturb the system variable of interest. Also, Baye's theorem can be readily applied for multiple measurements in sequence or multivariate conditioning.

Quantum systems: For both classical and quantum systems the state of the system allows one to compute the probability of future measurement outcomes. In classical mechanics it is possible, in principle, to have a state of zero uncertainty where the outcome of all measurements can be known with certainty in advance. In quantum mechanics however such a state cannot exist in principle and a state of maximal knowledge will still have uncertain measurement outcomes.

We describe the process of quantum measurement as coupling the system of interest with initial density matrix ρ to an ancillary meter system in a pure state $|\psi\rangle$ with the unitary U. An ideal measurement is then performed on the meter to infer information about the state of the system, which is modelled by projecting the meter onto $\langle q|$, where q is the measurement outcome. The measurement operator or Kraus operator [2] that acts on the system subspace is $\Upsilon = \langle q| U |\psi\rangle$ and the conditional state is computed by

$$\rho_{\text{out}} = \frac{\Upsilon \rho_{\text{in}} \Upsilon^\dagger}{\Pr(q)}, \tag{2}$$

where the subscripts indicate the initial and output state and

$$\Pr(q) = \text{Tr}\left\{\Upsilon^\dagger \Upsilon \rho_{\text{in}}\right\}, \tag{3}$$

is the probability of obtaining the measurement outcome q. For an Υ diagonal in the system variable of interest X we may write

$$\langle X| \rho_{\text{out}} |X\rangle = \frac{\Upsilon^\dagger(X)\Upsilon(X) \langle X| \rho_{\text{in}} |X\rangle}{\Pr(q)}. \tag{4}$$

Note the similarity of this expression to the classical Baye's rule Eq. (1) where $\Upsilon^\dagger(X)\Upsilon(X)$ is the noise distribution on the meter for a given X of the system.

Finally, we would like to note that taking a continuum limit of repeated application of Eq. (2) can be used to describe continuous measurement, which can be cast in the form of a stochastic master equation. The reader is directed to Ref. [1] for a more detailed derivation of the master equation.

Supplementary Note 2: Model of the Optomechanical System

In the following section we begin with a Hamiltonian of the canonical optomechanical form. This Hamiltonian is generally applicable to a variety of systems, including phononic crystals, trapped cold atoms, electromechanical

systems as well as the evanescently coupled system employed here. The Hamiltonian can be written in the following form:

$$H/\hbar = g_0 a^\dagger a(b^\dagger + b) + \Omega b^\dagger b + \Delta a^\dagger a \ , \tag{5}$$

where the Hamiltonian has been transformed into the rotating reference frame of the light by the unitary $U = e^{i\omega a^\dagger a t}$, with $H \to U(H - i\hbar\frac{\partial}{\partial t})U^\dagger$ and Δ is the detuning between the drive and the cavity frequency. The Heisenberg equations of motion, $\frac{\partial}{\partial t}\mathcal{O} = -\frac{i}{\hbar}[\mathcal{O}, H]$, can be then be written down for the optical annihilation operator and mechanical position and momentum operators respectively. A canonical addition of dissipation terms is then used to obtain the Langevin equation for the intra-cavity optical field, i.e.

$$\dot{a}(t) = -(\kappa + \gamma)a + i\Delta a - i\sqrt{2}g_0 a X_{\mathrm{M}} + \sqrt{2\kappa}a_{in} + \sqrt{2\gamma}\xi_\gamma \ . \tag{6}$$

Similarly, the mechanical dynamics are

$$\dot{P}_{\mathrm{M}}(t) = -\Omega X_{\mathrm{M}} - \Gamma P_{\mathrm{M}} - \sqrt{2}g_0 a^\dagger a + \sqrt{2\Gamma}\xi_P \ , \tag{7}$$

$$\dot{X}_{\mathrm{M}}(t) = \ \ \Omega P_{\mathrm{M}} \ , \tag{8}$$

where the position and momentum operators have been defined as $X_{\mathrm{M}}(t) = (b^\dagger + b)/\sqrt{2}$ and $P_{\mathrm{M}}(t) = i(b^\dagger - b)/\sqrt{2}$, with $[X_{\mathrm{M}}, P_{\mathrm{M}}] = i$. Note that κ and γ are optical amplitude decay rates, whilst Γ is the mechanical energy decay rate.

Optical dynamics: An essential requirement for the protocol used here is that the system be in the bad-cavity limit. In this limit, the optical measurement can directly probe the motion of the mechanical oscillator. This limit eliminates assumes fast changes in the field operator are negligible and hence the approximate solution is obtained as $\dot{a} \to 0$, i.e.

$$0 = -(\kappa + \gamma)a + i\Delta a - i\sqrt{2}g_0 a X_{\mathrm{M}} + \sqrt{2\kappa}a_{in} + \sqrt{2\gamma}\xi_\gamma \ , \tag{9}$$

which we can solve for the intra-cavity field

$$a(t) = \frac{\sqrt{2\kappa}a_{in} + \sqrt{2\gamma}\xi_\gamma}{(\kappa + \gamma) - i(\Delta - \sqrt{2}g_0 X_{\mathrm{M}})} \ . \tag{10}$$

We now consider a coherent drive applied to the optical input. The resultant state can be described by replacing $a_{in} \to \bar{a}_{in} + \xi_\kappa$, where $\bar{a}_{in}$ is the mean optical driving field. Then we have

$$a(t) = \frac{1}{\kappa + \gamma}\frac{\sqrt{2\kappa}\bar{a}_{in} + \sqrt{2\kappa}\xi_\kappa + \sqrt{2\gamma}\xi_\gamma}{1 + i\theta - i\delta} \ , \tag{11}$$

with the mean mechanical position $\bar{X}_{\mathrm{M}}$ introduced in a similar fashion as $X_{\mathrm{M}} \to X_{\mathrm{M}} + \bar{X}_{\mathrm{M}}$ and the new non-dimensional parameters defined as $\theta = \lambda X_{\mathrm{M}}$; $\lambda = \sqrt{2}g_0/(\kappa + \gamma)$, and $\delta = \Delta/(\kappa + \gamma) - \lambda\bar{X}_M$. The operators ξ_κ and ξ_γ are vacuum noise operators, provided the driving field is in a coherent state.

The steady-state intracavity photon number is now introduced. From Eq. (11), in the absence of any fluctuating terms, we can write the mean intracavity photon number, N, as

$$N = |\bar{a}|^2 = \frac{2\kappa}{(\gamma + \kappa)^2}\frac{|\bar{a}_{in}|^2}{1 + \delta^2} \ . \tag{12}$$

Replacing the mean driving field in Eq. (11) with N we have

$$a(t) \simeq \frac{\sqrt{N(1 + \delta^2)}}{1 + i\theta - i\delta} + \frac{1}{\kappa + \gamma}\frac{\sqrt{2\kappa}\xi_\kappa + \sqrt{2\gamma}\xi_\gamma}{1 - i\delta} \ . \tag{13}$$

Note we have assumed that the mechanical mixing with the vacuum noise operators ξ_κ and ξ_γ is negligible compared to the noise generated from the coherent term. We now choose the laser detuning, such that $\delta \equiv 0$, and expand the optical field about the operator θ

$$a(t) \simeq \sqrt{N}\left(1 - i\theta - \theta^2 + i\theta^3 + \theta^4 + ...\right) + \Xi \ , \tag{14}$$

where, for clarity, the vacuum noise terms have been subsumed into a single operator Ξ, i.e.

$$\Xi(t) = \frac{\sqrt{2\kappa}\xi_\kappa + \sqrt{2\gamma}\xi_\gamma}{\kappa+\gamma} \ . \tag{15}$$

It is clear from the series Eq. (14) that higher harmonics of the mechanical motion are present in the intracavity field fluctuations. The power series does not necessarily converge for all values of θ. In fact it is necessary to ensure that $\lambda^2\langle X_{\mathrm{M}}^2\rangle < 1$ must be satisfied. This can be understood as a requirement that the mechanical position fluctuations do not change the cavity resonance by more than κ. There after the output field can be written

$$\begin{aligned} a_{out}(t) &= a_{in} - \sqrt{2\kappa}a \ , \\ &= a_{in}\left(1 - \frac{2\kappa}{\gamma+\kappa}\frac{1}{1+i\theta}\right) - \sqrt{2\kappa}\ \Xi \ , \\ &\simeq a_{in}\left(\frac{\gamma-\kappa}{\gamma+\kappa} + \frac{2\kappa}{\gamma+\kappa}(i\theta+\theta^2-i\theta^3+...)\right) - \sqrt{2\kappa}\ \Xi \ . \end{aligned} \tag{16}$$

Replacing a_{in} with the mean field plus fluctuations and collecting terms, and dropping the products of noise terms, we can write Eq. (16) as

$$\begin{aligned} a_{out}(t) =& \sqrt{\frac{N}{2\kappa}}(\gamma-\kappa) + \frac{\gamma-\kappa}{\gamma+\kappa}\xi_\kappa - \frac{\sqrt{4\kappa\gamma}}{\gamma+\kappa}\xi_\gamma + \\ & \sqrt{2\kappa N}(i\lambda X_{\mathrm{M}} + \lambda^2 X_{\mathrm{M}}^2 - i\lambda^3 X_{\mathrm{M}}^3 + ...) \end{aligned} \tag{17}$$

The four terms in Eq. (17) can be identified respectively as the mean field term; the laser noise term; the cavity loss noise term; and the mechanical motion terms.

This field is then detected in the experiment using a homodyne with finite detection efficiency. This can be modelled as a beam-splitter that mixes vacuum with the signal before detection, i.e. $a_{det} = \sqrt{\eta}\ a_{out} + \sqrt{1-\eta}\ \xi_b$ where it is noted the vacuum noise operators ξ_a and ξ_b are not correlated. The homodyne then measures an arbitrary quadrature of the light, i.e.

$$i_\phi(t) = (a_{det}e^{-i\phi} + a_{det}^\dagger e^{i\phi})/\sqrt{2} \ , \tag{18}$$

and so the detected signal, given the output field can be written

$$\begin{aligned} i_\phi(t) =& \frac{e^{-i\phi}}{\sqrt{2}}\left[\sqrt{1-\eta}\ \xi_b \ + \sqrt{\eta}\left(\frac{\gamma-\kappa}{\gamma+\kappa}\xi_\kappa - \frac{\sqrt{4\kappa\gamma}}{\gamma+\kappa}\xi_\gamma\right)\right] \\ &+ \ \mathrm{h.c.} \\ &+ \ 2\sqrt{\eta\kappa N}\cos\phi\left(\frac{\gamma-\kappa}{2\kappa} + \lambda^2 X_{\mathrm{M}}^2 + \ ...\right) \\ &+ \ 2\sqrt{\eta\kappa N}\sin\phi\Big(\lambda X_{\mathrm{M}} - \lambda^3 X_{\mathrm{M}}^3 + \ ...\Big) \ . \end{aligned} \tag{19}$$

The detected field for for the specific cases of $\phi = 0$ and $\phi = \pi/2$ give the optical output amplitude ($X_{\mathrm{L}}^{\mathrm{out}}$) and phase ($P_{\mathrm{L}}^{\mathrm{out}}$) quadratures respectively with

$$\begin{aligned} X_{\mathrm{L}}^{\mathrm{out}}(t) = & 2\sqrt{\eta\kappa N}\left[\frac{\gamma-\kappa}{2\kappa} + \lambda^2 X_{\mathrm{M}}^2 + ...\right] \\ & + \sqrt{\eta}\left(\frac{\gamma-\kappa}{\gamma+\kappa}X_\xi^\kappa - \frac{\sqrt{4\kappa\gamma}}{\gamma+\kappa}X_\xi^\gamma\right) + \sqrt{1-\eta}X_\xi^b \ , \end{aligned} \tag{20}$$

and

$$\begin{aligned} P_{\mathrm{L}}^{\mathrm{out}}(t) = & 2\sqrt{\eta\kappa N}\left[\lambda X_{\mathrm{M}} - \lambda^3 X_{\mathrm{M}}^3 + ...\right] \\ & + \sqrt{\eta}\left(\frac{\gamma-\kappa}{\gamma+\kappa}P_\xi^\kappa - \frac{\sqrt{4\kappa\gamma}}{\gamma+\kappa}P_\xi^\gamma\right) + \sqrt{1-\eta}P_\xi^b \ . \end{aligned} \tag{21}$$

Importantly, it can be seen that the two optical quadratures carry information about the even and odd powers of the mechanical position on the amplitude and phase respectively.

Returning to the arbitrary quadrature representation, the resulting mean-subtracted correlation function (assuming X_M is zero-mean and ξ_a; ξ_b; a_{in} are all vacuum noise operators), can be written in the Fourier domain as

$$\begin{aligned}
\langle i_\phi(\omega) i_\phi(\omega')\rangle =& \left[1 - \eta\mu_1 \sin(2\phi)\left(\chi_\mathrm{M}(\omega) + \chi_\mathrm{M}(\omega')\right)\right] \times \left\langle \xi(\omega)\xi^\dagger(-\omega') + \xi^\dagger(-\omega)\xi(\omega')\right\rangle \\
&+ 2\eta\mu_1 \sin^2(\phi) \left\langle X_\mathrm{M}(\omega) X_\mathrm{M}(\omega')\right\rangle \\
&+ 2\eta\mu_2 \cos^2(\phi) \left\langle X^2_\mathrm{M}(\omega) X^2_\mathrm{M}(\omega')\right\rangle \\
&+ 2\eta\sqrt{\mu_1\mu_2}\,\frac{\sin(2\phi)}{2}\left[\left\langle X_\mathrm{M}(\omega) X^2_\mathrm{M}(\omega')\right\rangle + \left\langle X^2_\mathrm{M}(\omega) X_\mathrm{M}(\omega')\right\rangle\right] \\
&- 2\eta\mu_2 \sin^2(\phi)\left[\left\langle X_\mathrm{M}(\omega) X^3_\mathrm{M}(\omega')\right\rangle + \left\langle X^3_\mathrm{M}(\omega) X_\mathrm{M}(\omega')\right\rangle\right] \\
&+ \mathcal{O}(\lambda^5)\ ,
\end{aligned} \tag{22}$$

where the constants μ_1 and μ_2 are

$$\mu_1 = 2\kappa N\lambda^2, \quad \text{and} \quad \mu_2 = 2\kappa N\lambda^4\ , \tag{23}$$

and the new vacuum noise operator ξ (which is a linear combination of previous vacuum noise operators) satisfies the property $\left\langle \xi(\omega)\xi^\dagger(-\omega') + \xi^\dagger(-\omega)\xi(\omega')\right\rangle = 2\pi\delta(\omega+\omega')$.

The first line of Eq. 22 contains terms that arise from the vacuum noise on the light and the correlation of the optical fluctuations with the linearised mechanical motion, where the mechanical response (detailed later) was used. Notably the correlation term is unobservable on the amplitude quadrature (i.e. $\phi = 0$), therefore need not be considered during amplitude quadrature measurements. The next line is the linearised mechanical fluctuations, followed by the fluctuations proportional to the quadratic motion of the mechanics. Identification of these two terms allows us to label μ_1 and μ_2 as the linear and quadratic measurement rates respectively. The remaining two terms are corrections (up to order λ^4) when measuring on neither the phase or amplitude quadrature.

Mechanical dynamics: Equations (7) & (8) may be transformed into the Fourier domain to more easily realise a solution, so that

$$-i\omega X_\mathrm{M}(\omega) = \Omega P_\mathrm{M}\ , \tag{24}$$

$$-i\omega P_\mathrm{M}(\omega) = -\,\Omega X_\mathrm{M} - \Gamma P_\mathrm{M} - 2G X_\mathrm{L} + \sqrt{2\Gamma}\xi_P\ , \tag{25}$$

where the optomechanical back-action term has been linearised as $g_0 a^\dagger a \to \sqrt{2}G X_\mathrm{L}$ with the mean-subtracted optical; (mechanical) amplitude $X_\mathrm{L}(\omega) = (a^\dagger(-\omega) + a(\omega))/\sqrt{2}$; $(X_\mathrm{M}(\omega) = (b^\dagger(-\omega) + b(\omega))/\sqrt{2})$ with $G = \sqrt{N}g_0$. It should be noted that this linearisation step is very well justified due to the large intracavity photon number and essentially classical state of the optical field. The nonlinearity of the measurement in the previous section is a significant and observable effect, whilst the nonlinearity of the back-action generated by this term can be safely treated linearly. The noise operator $\xi_\mathrm{P}(t)$ can be shown to satisfy the property: $\langle \xi_P(\omega)\xi_P(\omega')\rangle_\mathrm{S} = 2\pi(\bar{n}+1/2)\delta(\omega+\omega')$, where the subscript S denotes the symmetrised expectation value, and $\bar{n}$ is the thermal occupation of the bath. The solution to these coupled equations can then be written as

$$X_\mathrm{M}(\omega) = \chi_\mathrm{M}\left(2G X_L - \sqrt{2\Gamma}\xi_P\right)\ , \tag{26}$$

where the mechanical response, χ, is given by

$$\chi_\mathrm{M}(\omega) = \frac{\Omega}{\omega^2 - \Omega^2 + i\Gamma\omega}\ . \tag{27}$$

The symmetrised mechanical correlation function for zero detuning is then

$$\begin{aligned}
&\langle X_\mathrm{M}(\omega) X_\mathrm{M}(\omega')\rangle_\mathrm{S} \\
&= \chi_\mathrm{M}(\omega)\chi_\mathrm{M}(\omega')\left(4G^2\langle X_L(\omega) X_L(\omega')\rangle_\mathrm{S} + 2\Gamma\langle \xi_\mathrm{P}(\omega)\xi_\mathrm{P}(\omega')\rangle_\mathrm{S}\right)\ , \\
&= \chi_\mathrm{M}(\omega)\chi_\mathrm{M}(\omega')\left(\frac{4G^2}{(\kappa+\gamma)} + 2\Gamma(\bar{n}+1/2)\right) 2\pi\delta(\omega+\omega')\ ,
\end{aligned} \tag{28}$$

where we have observed that the correlation function of the mean-subtracted amplitude quadrature can be approximated (neglecting the non-linear mechanical contributions) by the vacuum noise fluctuations

$$\begin{aligned}
\langle X_L(\omega)X_L(\omega')\rangle \approx& \frac{1}{2}\left\langle \Xi(\omega)\Xi^\dagger(\omega') + \Xi^\dagger(\omega)\Xi(\omega')\right\rangle \ , \\
=& \frac{\kappa\left\langle \xi_\kappa(\omega)\xi_\kappa^\dagger(-\omega') + \xi_\kappa^\dagger(-\omega)\xi_\kappa(\omega')\right\rangle}{(\kappa+\gamma)^2} \\
&+ \frac{\gamma\left\langle \xi_a(\omega)\xi_a^\dagger(-\omega') + \xi_a^\dagger(-\omega)\xi_a(\omega')\right\rangle}{(\kappa+\gamma)^2} \ , \\
=& \frac{2\pi\delta(\omega+\omega')}{(\kappa+\gamma)} \ .
\end{aligned} \tag{29}$$

The symmetrised mechanical power spectrum is then

$$\begin{aligned}
S_{X_\mathrm{M}X_\mathrm{M}}(\omega) =& \frac{1}{2\pi}\int d\omega' \left\langle X_\mathrm{M}(\omega)X_\mathrm{M}(\omega')\right\rangle_\mathrm{S} \ , \\
=& |\chi_\mathrm{M}(\omega)|^2\left(\frac{4G^2}{(\kappa+\gamma)} + 2\Gamma(\bar{n}+1/2)\right) \ ,
\end{aligned} \tag{30}$$

with the modulus-square of the response function given by

$$|\chi_\mathrm{M}(\omega)|^2 = \frac{\Omega^2}{(\omega^2-\Omega^2)^2+\Gamma^2\omega^2} \ . \tag{31}$$

This function has poles at $\omega \approx \pm(\Omega \pm i\Gamma/2)$, giving rise to two peaks in the spectral density at $\pm\Omega$, with linewidth of Γ. Additionally, the integral over all frequencies is proportional to the linewidth, that is, $\int |\chi_\mathrm{M}|^2 d\omega = \pi/\Gamma$. The mechanical variance is found by integrating over ω, i.e.

$$\begin{aligned}
\left\langle X_\mathrm{M}^2\right\rangle =& \frac{1}{2\pi}\int d\omega S_{X_\mathrm{M}X_\mathrm{M}}(\omega) \ , \\
=& \frac{2G^2}{\Gamma(\kappa+\gamma)} + (\bar{n}+1/2) \ .
\end{aligned} \tag{32}$$

It follows that if the (dimensioned) mechanical variance is known, the thermal occupation (in the bath-dominated regime) is simply

$$\bar{n} + \frac{1}{2} = \frac{\left\langle x_\mathrm{M}^2\right\rangle}{2\ x_0^2} \ , \tag{33}$$

where $x_0 = \sqrt{\frac{\hbar}{2m\omega_\mathrm{M}}}$ is the mechanical zero-point motion.

Supplementary Note 3: Spectral Characteristics of the Nonlinear Measurement

Power spectrum of the quadratic motion: From Eq. (22), we see the photocurrent contains a term which is directly proportional to the X_M^2 correlation function. The properties of the mechanical X_M^2 correlation function may be computed via

$$\begin{aligned}
&\left\langle X_\mathrm{M}^2(\omega)X_\mathrm{M}^2(\omega')\right\rangle = \\
&\left\langle \frac{1}{(2\pi)^2}\int X_\mathrm{M}(\omega-f)X_\mathrm{M}(f)\ df \int X_\mathrm{M}(\omega'-g)X_\mathrm{M}(g)\ dg\right\rangle \ ,
\end{aligned} \tag{34}$$

where we have made use of the convolution theorem. This expression is related to the mechanical response function and noise operator via

$$\begin{aligned}
&\left\langle X_\mathrm{M}^2(\omega)X_\mathrm{M}^2(\omega')\right\rangle = \\
&\frac{1}{(2\pi)^2}\int\int df dg\ \chi_\mathrm{M}(f)\chi_\mathrm{M}(\omega-f)\chi_\mathrm{M}(g)\chi_\mathrm{M}(\omega'-g) \\
&\quad\times\left(4\Gamma^2\left\langle \xi_P(f)\xi_P(g)\xi_P(\omega-f)\xi_P(\omega'-g)\right\rangle\right) \ .
\end{aligned} \tag{35}$$

Using the fact that the ξ operators are Gaussian, we can use the Isserlis-Wick theorem to rewrite the noise correlation, i.e.

$$\begin{aligned} &\langle \xi_P(f)\xi_P(g)\xi_P(\omega-f)\xi_P(\omega'-g)\rangle_{\rm S} = \\ &\quad (2\pi)^2(\bar{n}+1/2)^2 \times \Big(\delta(f+g)\delta(\omega+\omega'-f-g) \\ &\qquad + \delta(f+\omega'-g)\delta(\omega-f+g) + \delta(\omega)\delta(\omega')\Big)\ . \end{aligned} \tag{36}$$

After performing the integral over f in the first two terms of the Isserlis-Wick expansion, we have

$$\begin{aligned} &\left\langle X_{\rm M}^2(\omega)X_{\rm M}^2(\omega')\right\rangle_{\rm S} = \left(4\Gamma^2(\bar{n}+1/2)^2\right) \times \\ &\Big(\int dg\chi_{\rm M}(-g)\chi_{\rm M}(\omega+g)\chi_{\rm M}(g)\chi_{\rm M}(\omega'-g)\delta(\omega+\omega') \\ &+ \int dg\chi_{\rm M}(-g)\chi_{\rm M}(\omega+g)\chi_{\rm M}(g)\chi_{\rm M}(\omega'-g)\delta(\omega+\omega') \\ &+ \int df\chi_{\rm M}(f)\chi_{\rm M}(\omega-f)\int dg\chi_{\rm M}(g)\chi_{\rm M}(\omega'-g)\delta(\omega)\delta(\omega')\Big)\ , \end{aligned} \tag{37}$$

from which we can obtain the symmetrised power spectrum

$$\begin{aligned} S_{X_{\rm M}^2X_{\rm M}^2}(\omega) =& \frac{1}{2\pi}\int d\omega'\left\langle X_{\rm M}^2(\omega)X_{\rm M}^2(\omega')\right\rangle_{\rm S} \\ =& \frac{1}{2\pi}\left(4\Gamma^2(\bar{n}+1/2)^2\right) \times \\ &\left(2\int d\omega'|\chi_{\rm M}(\omega')|^2|\chi_{\rm M}(\omega-\omega')|^2 + \left[\int d\omega'|\chi_{\rm M}(\omega')|^2\right]^2\delta(\omega)\right)\ , \end{aligned} \tag{38}$$

where the symmetry property of the mechanical response, i.e. $\chi_{\rm M}(\omega) = \chi_{\rm M}^\star(-\omega)$ has been used. Using the convolution operator $(*)$, and the normalisation of $\chi_{\rm M}$, Eq. (38) can be more simply written as

$$\begin{aligned} S_{X_{\rm M}^2X_{\rm M}^2}(\omega) =& \frac{1}{2\pi}\left(4\Gamma^2(\bar{n}+1/2)^2\right) \times \\ &\left(2\ |\chi_{\rm M}(\omega)|^2 * |\chi_{\rm M}(\omega)|^2 + \frac{\pi^2}{\Gamma^2}\delta(\omega)\right)\ . \end{aligned} \tag{39}$$

The convolution in Eq. (39) can then be found analytically

$$\begin{aligned} &|\chi_{\rm M}(\omega)|^2 * |\chi_{\rm M}(\omega)|^2 = \\ &\qquad \frac{2\pi\Omega^2\left(4\Gamma^2+\omega^2+4\Omega^2\right)}{\Gamma\left(\Gamma^2+\omega^2\right)\left(4\Gamma^2\omega^2+\left(\omega^2-4\Omega^2\right)^2\right)}\ . \end{aligned} \tag{40}$$

This equation has poles at $\omega = \pm i\Gamma$ and $\omega \approx \pm(2\Omega \pm i\Gamma)$, thus giving rise to peaks in the spectral density at zero frequency, and $\pm 2\Omega$ (in the high-Q limit). Also note that the linewidth of the X^2 spectrum at all 3 peaks is equal to 2Γ. Equation (40) has the normalisation property $\int d\omega\ |\chi_{\rm M}(\omega)|^2 * |\chi_{\rm M}(\omega)|^2 = \pi^2/\Gamma^2$. The mechanical position squared variance may also then be computed

$$\begin{aligned} \left\langle X_{\rm M}^2X_{\rm M}^2\right\rangle &= \frac{1}{2\pi}\int d\omega\ S_{X^2X^2}(\omega) \\ &= \frac{1}{(2\pi)^2}\left(4\Gamma^2(\bar{n}+1/2)^2\right) \times \frac{3\pi^2}{\Gamma^2}\ , \\ &= 3(\bar{n}+1/2)^2\ , \end{aligned} \tag{41}$$

which satisfactorily in agreement with the elementary result used later in Eq. (47).

Power in higher harmonics: Of experimental interest is the power generated in each harmonic of the mechanical oscillation oscillation frequency by the optical nonlinearity. Making use of the small $\lambda^2\langle X_{\rm M}^2\rangle$ approximation from

earlier, we need only consider the contributions of the nth power of $X_{\rm M}$ to the nth harmonic. Mathematically, we are considering only the leading term in the nth power expansion of a harmonic function

$$\begin{aligned} \cos^n\theta &= \frac{1}{2^n}(e^{i\theta}+e^{-i\theta})^n \ , \\ &= \frac{1}{2^{n-1}}(\cos(n\theta)+...) \ . \end{aligned} \tag{42}$$

We would like to know what fraction of the total power is present in the leading term, which can be found by squaring both sides and integrating over one cycle. For the total scaled-power in the nth moment of the mechanical position, we have

$$\left\langle (X_{\rm M}^n)^2\right\rangle \propto p_{tot}(n) = \frac{1}{2\pi}\int_0^{2\pi}\cos^{2n}(\theta)d\theta \tag{43}$$

This integral can be found using a recursion formula, resulting in the product

$$\begin{aligned} p_{tot}(n) &= \prod_{k=0}^{n-1}\frac{2k+1}{2k+2} \ , \\ &= \frac{(2n)!}{2^{2n}(n!)^2} \ . \end{aligned} \tag{44}$$

The scaled-power in the nth harmonic of the nth moment is simply given by

$$\begin{aligned} p_{n\omega_{\rm M}}(n) &= \frac{1}{2^{2n-2}}\frac{1}{2\pi}\int_0^{2\pi}\cos^2(n\theta)d\theta \ , \\ &= \frac{1}{2^{2n-1}} \ . \end{aligned} \tag{45}$$

As a result, the fraction of the total power present in the nth harmonic is given by

$$f_n = \frac{p_{n\omega_{\rm M}}(n)}{p_{tot}(n)} = \frac{2(n!)^2}{(2n)!} \ . \tag{46}$$

If the fluctuations of the mechanical position are assumed Gaussian, then the total power in the nth moment can be expressed in terms of the mechanical variance using the Isserlis-Wick theorem

$$\langle (X_{\rm M}^n)^2\rangle = \frac{(2n)!}{2^n n!}\langle X_{\rm M}^2\rangle^n \ , \tag{47}$$

and so the mechanical power ($p_{\rm M}$) in the nth harmonic from the nth moment can be written

$$p_{\rm M}(n) = f_n\langle (X_{\rm M}^n)^2\rangle = \frac{n!}{2^{n-1}}\langle X_{\rm M}^2\rangle^n \ . \tag{48}$$

Neglecting the radiation pressure back-action, the powers in each harmonic are proportional to

$$p_{\rm M}(n) = \frac{n!}{2^{n-1}}(\bar{n}+1/2)^n \ . \tag{49}$$

The respective optical power ($p_{\rm L}$) scales as λ^{2n} times the mechanical power when measuring on the phase quadrature for odd values of n and the amplitude quadrature for even values of n, i.e.

$$p_{\rm L}(n) \propto \frac{\lambda^{2n}n!}{2^{n-1}}(\bar{n}+1/2)^n \ . \tag{50}$$

The first few terms for large thermal occupation are tabulated below.

freq.:	$\omega_{\rm M}$	:	$2\omega_{\rm M}$	:	$3\omega_{\rm M}$	:	$4\omega_{\rm M}$
$p_{\rm M}$:	$f_1\langle X_{\rm M}^2\rangle$	:	$f_2\langle X_{\rm M}^4\rangle$	:	$f_3\langle X_{\rm M}^6\rangle$	:	$f_4\langle X_{\rm M}^8\rangle$
	$\bar{n}$	:	$\bar{n}^2$	:	$\frac{3}{2}\bar{n}^3$	:	$3\bar{n}^4$
$p_{\rm L}$:	$f_1\lambda^2\langle X_{\rm M}^2\rangle$	:	$f_2\lambda^4\langle X_{\rm M}^4\rangle$	:	$f_3\lambda^6\langle X_{\rm M}^6\rangle$	:	$f_4\lambda^8\langle X_{\rm M}^8\rangle$
	$\lambda^2\bar{n}$	:	$\lambda^4\bar{n}^2$	:	$\frac{3}{2}\lambda^6\bar{n}^3$	:	$3\lambda^8\bar{n}^4$

Note, although the fluctuations at $\omega_{\rm M}$ are Gaussian, the higher harmonics, due to the fact they are generated via a nonlinear transformation of Gaussian fluctuations, will themselves be non-Gaussian.

Using Stirling's approximation, we can compute the scaling of the power in the higher harmonics for very large n, i.e.

$$\ln(P_{\rm M}(n)) \approx n\ln(n) + \mathcal{O}(n\ln(\lambda^2\bar{n})) \tag{51}$$

Curiously, this expression is *always* divergent for large n irrespective of the magnitude of $\lambda^2\bar{n}$. This is due to the fact that the power-series Eq. (14) only converges for $|\theta| < 1$. Since this expansion is operator valued, this condition is not guaranteed. In spite of the generally non-convergence behaviour of this series, in practice it is possible to use the result obtained for the first few orders with quantitative accuracy in the limit of small $\lambda^2\bar{n}$.

Supplementary Note 4: Spectral Filtering and Quadrature estimation

From Eq. (19), the measured photocurrent when the homodyne angle is set to $\pi/4$ is of the following form:

$$i_{\phi=\pi/4}(t) = \sqrt{2\eta\kappa N}\left(\lambda X_{\rm M} + \lambda^2 X_{\rm M}^2\right) + \text{Noise}\ , \tag{52}$$

and consequently is a linear combination of the linear and quadratic measurements of mechanical motion. Since, as discussed earlier, the linear and quadratic components are separated spectrally, we can in principle recover simultaneous information about both the quadratic motion and the linear motion of the mechanics by appropriate spectral filtering. Writing the mechanical motion in terms of the mechanical postion quadratures, X and Y,

$$X_{\rm M}(t) = X(t)\cos\omega_{\rm M}t + Y(t)\sin\omega_{\rm M}t\ , \tag{53}$$

we can see that the quadratures of motion may be recovered (in the high-Q limit) by multiplying by a sin or cos function (at $\omega_{\rm M}$) and integrating with respect to time. The quadratic displacement can be written in terms of the mechanical position quadrature as

$$X_{\rm M}^2 = \ \tfrac{1}{2}(X^2+Y^2)\ +\ \tfrac{1}{2}(X^2-Y^2)\cos(2\omega_{\rm M}t) +\ \tfrac{1}{2}(XY+YX)\sin(2\omega_{\rm M}t)\ , \tag{54}$$

where it is clear the displacement-squared quadratures of motion may be defined as

$$P = \tfrac{1}{2}(X^2-Y^2); \qquad Q = \tfrac{1}{2}(XY+YX)\ . \tag{55}$$

Similarly, the quadratic motion position quadratures may be recovered by integrating with sin and cos functions respectively at twice the mechanical resonance frequency.

To emphasise the preparation aspect of the protocol, we separate the dataset at some time origin t, into purely causal and purely acausal data, that is data that was acquired at $t' < t$ and at $t' > t$, respectively. Since the causal data and acausal data are independent upon each other (but highly correlated due to the mechanical motion), the causally-prepared conditional state at time t may be verified with the independent acausal linear measurements made after time t. Here the state verification will amount to measuring the two mechanical position quadratures, and reconstructing a point in phase-space.

State reconstruction is performed by firstly scaling the photocurrent signal i_ϕ to produce an estimate of $X_{\rm M}$, specifically the estimate is given from the photocurrent by $\widetilde{X}_{\rm M} = i_\phi/\lambda\sqrt{2\eta\kappa N}$. The signal at ω_M is then filtered and decomposed into quadratures using the following transformations

$$\begin{aligned}\tilde{X}(t) &= \int_0^\infty \frac{dt'}{\tau}\cos[\omega_M(t+t')]\ e^{-t'/2\tau}\widetilde{X}_{\rm M}(t+t')\ ,\\ \tilde{Y}(t) &= \int_0^\infty \frac{dt'}{\tau}\sin[\omega_M(t+t')]\ e^{-t'/2\tau}\widetilde{X}_{\rm M}(t+t')\ ,\end{aligned} \tag{56}$$

where τ is inversely proportional to the filter bandwidth. The Fourier transform of this filter has a power spectrum that is Lorentzian, and hence only filters frequency components near a frequency $\omega_{\rm M}$. We note that in the limit as $\tau \to \infty$, the filter approaches the definition of the acausal position quadratures. Similarly, we can rescale the photocurrent to obtain an estimate of $X_{\rm M}^2$ as $\widetilde{X}_{\rm M}^2 = i_\phi/\lambda^2\sqrt{2\eta\kappa N}$ and define the causal filter that acts on the $2\omega_{\rm M}$

component of the signal, i.e.

$$\tilde{P}(t) = \int_0^\infty \frac{dt'}{\tau} \cos[2\omega_M(t-t')]\; e^{-t'/2\tau} \widetilde{X}^2_{\mathrm{M}}(t-t')\ ,$$
$$\tilde{Q}(t) = \int_0^\infty \frac{dt'}{\tau} \sin[2\omega_M(t-t')]\; e^{-t'/2\tau} \widetilde{X}^2_{\mathrm{M}}(t-t')\ . \tag{57}$$

In practice these convolutions are performed in the Fourier domain for computational efficiency.

Supplementary Note 5: Conditioning Protocol and Reconstruction

For ease of demonstration of the conditioning protocol in a classical regime, simultaneous and continuous measurement of both quadratures of motion at both the mechanical frequency and its second harmonic is performed. The photocurrent is digitised into discrete samples, and for each sample, the corresponding values of $\tilde{X}$; $\tilde{Y}$; $\tilde{P}$ and $\tilde{Q}$ are computed using the discrete forms of Eq. (56-57). In practice, the simplest way to perform the conditional rotations of the linear data (described in the main text) is with complex arithmetic. The complex numbers $c = \tilde{P} + i\tilde{Q}$ (conditioning vector) and $r = \tilde{X} + i\tilde{Y}$ (read-out vector) are computed, and then r is transformed according to

$$r \to r\sqrt{c^*/|c|}\ . \tag{58}$$

This non-linear transformation has the effect of rotating the linear data into the X direction in phase space, conditioned upon the outcome of the quadratic measurement. Additionally, a conditioning operation is performed on the value of $2|c|$, that is to say, only values within some small range of a desired value C are kept: $(C - \epsilon) < 2|c| < (C + \epsilon)$. Typically ϵ is chosen to be on the order of half the measurement uncertainty. This operation further localises the state into two small regions of phase space at $X = \pm\sqrt{C}$. The application of these two steps on actual experimental data is illustrated in Fig. 2.

In a quantum regime, it should be noted that both X and Y; (P and Q) do not commute, since the mechanical position; (position squared) does not commute with itself at different times. This will introduce an additional shot-noise penalty in the measurement, which although not precluding the preparation of quantum non-Gaussian states (see Sec. 10), will however preclude quantum state reconstruction using the record of $X(t)$ and $Y(t)$. Additionally, as discussed in Sec. 7 nonlinear conditioning and linear read-out cannot occur simultaneously, as the linear read-out prevents complete cancellation of the linearised back-action on the mechanics. Therefore in a quantum regime, separate periods of state conditioning using pure X^2_{M} mesurement, followed by a period of quantum state reconstruction (that could for example be implemented using a quantum non-demolition measurement of arbitrary quadratures of the mechanical motion), will be required.

Supplementary Note 6: Requirements for Quantum Regime of Nonlinear Measurement

In order to ensure the protocol is able to generate states in a quantum regime, the nonlinear measurement rate must exceed all decoherence rates. A most basic requirement then is that decoherence arising from interaction of the mechanical oscillator with the thermal bath be negligible compared with the non-linear measurement rate. As identified from Eq. (22), the rate at which the system is conditioned by the quadratic measurement is for a pure amplitude quadrature meausrement ($\phi = 0$) is given by

$$\eta\mu_2 = 2\eta\kappa N\lambda^4 = \zeta^4\eta\frac{8G^2g_0^2}{\kappa^3}\ , \tag{59}$$

where it is convenient to define the cavity escape efficiency $\zeta = \kappa/(\kappa + \gamma)$. From Eq. (28), we can identify the two decoherence (noise) process acting on the mechanical oscillator as the optical back-action and the thermal bath, with respective heating rates defined as

$$\mu_{\mathrm{BA}} = \zeta\frac{4G^2}{\kappa}\ , \tag{60}$$
$$\mu_{\mathrm{Env}} = 2\Gamma(\bar{n} + 1/2)\ . \tag{61}$$

Considering the case where the bath noise is dominant, the measurement rate should exceed the bath decoherence, i.e. $\eta\mu_2 > \mu_{\mathrm{Env}}$, i.e.

$$\zeta^4\eta\frac{2G^2}{\Gamma\kappa}\frac{2g_0^2}{\kappa^2} > \bar{n} + 1/2\ . \tag{62}$$

The left hand side of the above equation can be thought of as a quadratic cooperativity, and must dominate the bath occupation for thermal noise to be considered negligible. However, the back-action noise arising from the optical interaction can at the same time be significant, and therefore cannot be neglected. We might naïvely expect that an amplitude quadrature measurement, described in Eq. (22) for $\phi = 0$ will produce a conditional state dominated by the quadratic measurement process, provided the quadratic measurement rate μ_2 dominates the decoherence rates of the mechanics, that is

$$\zeta^4 \eta \frac{4G^2}{\kappa} \frac{2g_0^2}{\kappa^2} > \left(\zeta \frac{4G^2}{\kappa} + 2\Gamma(\bar{n} + 1/2) \right) . \tag{63}$$

In the high-cooperativity regime, this requirement is reduced to

$$\zeta^2 \lambda^2 > \frac{1}{\zeta \eta} . \tag{64}$$

This seems to imply, similar to the membrane in the middle scheme, a single photon strong coupling regime ($\lambda > 1$) is required. However, as shown in the next section, this requirement can be substantially relaxed using feedback.

Supplementary Note 7: Feedback Suppression of the Linear Back-Action

In the previous section, the requirement (64) can be thought of as arising from the competition between the nonlinear measurement, and the noise added to the mechanics by the radiation-pressure interaction. However, since the radiation pressure noise on the mechanical oscillator is strongly correlated with fluctuations on the optical amplitude quadrature around the mechanical resonance frequency, and this information is readily available in the homodyne photocurrent, it is in principle possible to completely remove the (linearised) radiation-pressure noise on the mechanics. This may be readily seen by explicitly utilitising the measurement record to cancel the back-action noise on the mechanics with feed-back.

We begin by introducing a feed-back signal F, such that Eq. (26) is modified as follows

$$X = \chi_{\mathrm{M}} \left(2GX_L - \sqrt{2\Gamma}\xi_P + F \right) , \tag{65}$$

and require that the signal F is sufficiently weakly coupled to the system that it may be treated classically. The feed-back force may in practice be implemented using an external actuator, for example a piezo-electric module attached to the nano-string substrate, or using radiation pressure itself by amplitude modulating the input optical drive. The latter technique however is an in-loop approach, and will result in the squashing of the detected amplitude quadrature noise.

The simplest choice of feed-back filter is to use an LTI process H acting on the homodyne measurement record of the optical amplitude quadrature, such that in the fourier domain the feed-back signal may be written: $F(\omega) = H(\omega)X_L(\omega)$. From the expression for the intracavity annihilation operator Eq. (14), the intracavity amplitude quadrature may be found. However the intracavity field fluctuations that drive noise on the mechanics are filtered by the mechanical response function. This means that only noise near the mechanical resonance frequency is responsible for the added variance to the mechanics. Hence we can make an approximation, and only consider noise terms that have significant spectral density near the mechanical resonance frequency. Since on the amplitude quadrature, the mechanical motion only generates fluctuations in the amplitude quadrature near twice the mechanical frequency (provided the oscillator has sufficiently high Q), we may safely neglect the mechanical contributions to the intracavity amplitude fluctuations. The component of the intracavity amplitude fluctuations with significant power near the mechanical resonance frequency given from the vacuum noise terms of Eq. (15)

$$X_L \approx X_\Xi = \frac{1}{\sqrt{2}}(\Xi + \Xi^\dagger) . \tag{66}$$

In the frequency band near the mechanical resonance frequency, the intracavity field is then perfectly correlated with a linear combination of the input noise operators. As a result it is convenient to relace the input noise operators with the intracavity field operator X_Ξ and a new field operator $\widetilde{X}_\Xi$ that gives zero covariance with X_Ξ, i.e. $\left\langle X_\Xi \widetilde{X}_\Xi \right\rangle = 0$. Written in terms of the input field operators from Eq. (15) we have

$$X_\Xi = \frac{1}{\kappa + \gamma} \left(\sqrt{2\kappa} X_\xi^\kappa + \sqrt{2\gamma} X_\xi^\gamma \right) , \tag{67}$$

$$\widetilde{X}_\Xi = \frac{1}{\kappa + \gamma} \left(\sqrt{2\gamma} X_\xi^\kappa - \sqrt{2\kappa} X_\xi^\gamma \right) . \tag{68}$$

Since the noise operators X^{κ}_{ξ} and X^{γ}_{ξ} have the same variance and are uncorrelated, the two new intracavity operators satisfy the zero covariance condition. Rearranging for the input noise operators we may write

$$X^{\kappa}_{\xi} = \frac{1}{2}\left(\sqrt{2\kappa}X_{\Xi} + \sqrt{2\gamma}\widetilde{X}_{\Xi}\right) , \tag{69}$$

$$X^{\gamma}_{\xi} = \frac{1}{2}\left(\sqrt{2\gamma}X_{\Xi} - \sqrt{2\kappa}\widetilde{X}_{\Xi}\right) . \tag{70}$$

Hence, we can replace the input field operators in Eq. (20) as follows

$$\begin{aligned} X^{\text{out}}_{\text{L}} &= 2\sqrt{\eta\kappa N}\left[\frac{\gamma-\kappa}{2\kappa} + \lambda^2 X^2_{\text{M}} + ...\right] \\ &+ \frac{\sqrt{\eta}}{2}\left(\sqrt{2\gamma}\widetilde{X}_{\Xi} - \sqrt{2\kappa}X_{\Xi}\right) + \sqrt{1-\eta}X^{b}_{\xi} . \end{aligned} \tag{71}$$

Since all the operators that appear in this expression are now uncorrelated, it becomes a simple matter to compute the Wiener-optimal filter. For an estimate of the quantity A from the measured quantity B the Wiener filter is given by

$$H(\omega) = \frac{S_{AB}(\omega)}{S_{BB}(\omega)} , \tag{72}$$

where S_{AB} is the cross-spectral density of the quantities A and B, and S_{BB} is the power spectrum of B. Here the measurement corresponds to $X^{\text{out}}_{\text{L}}$ and the desired estimate is the radiation pressure noise driving term in Eq. (26), that is $2GX_{\text{L}}$. We then have

$$H(\omega) = \frac{\sqrt{2\eta\kappa}G\int d\omega' \left\langle X_{\Xi}(\omega)X_{\Xi}(\omega')\right\rangle}{\frac{\eta\kappa}{2}\int d\omega' \left\langle X_{\Xi}(\omega)X_{\Xi}(\omega')\right\rangle + \frac{\eta\gamma}{2}\int d\omega' \left\langle \widetilde{X}_{\Xi}(\omega)\widetilde{X}_{\Xi}(\omega')\right\rangle + (1-\eta)\int d\omega' \left\langle X^{b}_{\xi}(\omega)X^{b}_{\xi}(\omega')\right\rangle} , \tag{73}$$

where the DC term and the mechanical quadratic motion spectral component of the detected signal have not been considered since they contribute negligible power near the mechanical resonance frequency ω_{M}. From Eq. (67) the variance of all the quantities in this expression can be written in terms of the vacuum field, i.e.

$$\begin{aligned} \left\langle \widetilde{X}_{\Xi}(\omega)\widetilde{X}_{\Xi}(\omega')\right\rangle = \left\langle X_{\Xi}(\omega)X_{\Xi}(\omega')\right\rangle = \\ \frac{2}{\kappa+\gamma}\left\langle X^{b}_{\xi}(\omega)X^{b}_{\xi}(\omega')\right\rangle \end{aligned} \tag{74}$$

Then the transfer function $H(\omega)$ then simply reduces to a frequency independent constant

$$H(\omega) = \zeta\eta\frac{4G}{\sqrt{2\eta\kappa}} , \tag{75}$$

with the cavity escape efficiency, $\zeta = \kappa/(\kappa+\gamma)$. We can compute the error that arises when this optimal feed-back is applied

$$\begin{aligned} \epsilon(\omega) &= 2GX_{L}(\omega) + H(\omega)X^{\text{out}}_{L}(\omega) \\ &\approx 2G(1-\zeta\eta)X_{\Xi} + \zeta\eta\frac{4G}{\sqrt{2\eta\kappa}}\left(\sqrt{2\gamma\eta}\widetilde{X}_{\Xi}/2 + \sqrt{1-\eta}X^{b}_{\xi}\right) , \end{aligned} \tag{76}$$

where we have approximated $X_{\text{L}} \approx X_{\Xi}$, and removed the mechanical contribution to $X^{\text{out}}_{\text{L}}$. The power spectrum of the error is then

$$\begin{aligned} S_{\epsilon\epsilon}(\omega) =& \int d\omega' \left\langle \epsilon(\omega)\epsilon(\omega')\right\rangle , \\ =&\left(4G^2(1-\zeta\eta)^2 2\zeta/\kappa + \zeta^2\eta^2\frac{16G^2}{2\eta\kappa}\left(\eta(1-\zeta) + (1-\eta)\right)\right)\times \\ &\int d\omega' \left\langle X^{b}_{\xi}(\omega)X^{b}_{\xi}(\omega')\right\rangle , \end{aligned} \tag{77}$$

where the result Eq. (74) has been used. The vacuum field is delta correlated, such that $\left\langle X^b_\xi(\omega)X^b_\xi(\omega')\right\rangle = \delta(\omega+\omega')/2$, and hence the integral can be collapsed to give the final form of the spectrum:

$$\begin{aligned} S_{\epsilon\epsilon}(\omega) =& \frac{4G^2}{\kappa}\zeta\left(\zeta\eta^2(1-\zeta)+\zeta\eta(1-\eta)+(1-\zeta\eta)^2\right) \ , \\ =& \frac{4G^2}{\kappa}\zeta\left(1-\zeta\eta\right) \ . \end{aligned} \tag{78}$$

The variance of this error term can be interpreted as the back-action heating rate of the mechanics in the presence of feed-back and is consistent with the no-feed-back case Eq. (60) when the limit $\eta \to 0$ is taken. Hence the back-action heating rate with feedback must have the following form

$$\mu_{\mathrm{BA}} = \frac{4G^2}{\kappa}\zeta(1-\zeta\eta) \ . \tag{79}$$

As might be expected, for perfect detection of the intracavity field ($\eta \to 1$; $\zeta \to 1$), the back-action may be perfectly cancelled. In a similar ad-hoc manner as before, we can compare the feed-back modified back-action heating rate to the quadratic measurement rate $\eta\mu_2$

$$\zeta^4\eta\frac{8G^2g_0^2}{\kappa^3} > \frac{4G^2}{\kappa}\zeta(1-\zeta\eta) \ , \tag{80}$$

which reduces to

$$\zeta^2\lambda^2 > \frac{(1-\zeta\eta)}{\zeta\eta} \ . \tag{81}$$

Thus by utilising the information contained in the amplitude quadrature around the mechanical resonance frequency, the single-photon strong coupling requirement is relaxed by a factor of $\sqrt{1-\zeta\eta}$.

This means, for example, with a 96% detection efficiency, and a cavity that is overcoupled by a factor of 10, the single-photon coupling rate need only be $\approx 1/3$ of the coupling rate required without feed-back with $g_0/\kappa = 0.3$, however as shown in the following section, even for substantially lower detection efficiency and/or coupling rate, negativity is still expected to arise.

Supplementary Note 8: Further Technical Requirements

Homodyne lock precision: The linearised OM interaction gives rise to phase shifts on the light generated by the mechanical motion. This means that in a spectral region around the mechanical resonance frequency, the fluctuations on the light are largely dominated by phase fluctuations. Considering the homodyne photocurrent, in the presence of some lock imprecision ξ, is given by:

$$i_\phi = \sin(\phi+\xi)P_{\mathrm{L}}^{\mathrm{out}} + \cos(\phi+\xi)X_{\mathrm{L}}^{\mathrm{out}} \tag{82}$$

Then if ξ is a Gaussian noise process, the detected photocurrent is:

$$\begin{aligned} \left\langle i_\phi^2\right\rangle \approx& \left\langle (P_{\mathrm{L}}^{\mathrm{out}})^2\right\rangle \sin^2(\phi)\left(1+\left\langle\xi^2\right\rangle\right) \\ &+ \left\langle (P_{\mathrm{L}}^{\mathrm{out}})^2\right\rangle \cos^2(\phi)\left\langle\xi^2\right\rangle \\ &+ \left\langle (X_{\mathrm{L}}^{\mathrm{out}})^2\right\rangle (...) \end{aligned} \tag{83}$$

Given the large magnitude of the ratio of the phase fluctuations to the shot noise in this experiment, we can safely neglect the contributions of the amplitude fluctuations near the mechanical resonance frequency, which are on the order of the optical shot noise. Then, at the point of maximum suppression, i.e. $\phi = 0°$, the detected signal is:

$$\left\langle i_{\phi=0°}^2\right\rangle = \left\langle i^2\right\rangle_{\max} = \left\langle (P_{\mathrm{L}}^{\mathrm{out}})^2\right\rangle \left\langle\xi^2\right\rangle \tag{84}$$

and at minimum suppression, i.e. $\phi = 90°$, the detected signal is:

$$\left\langle i_{\phi=90°}^2\right\rangle = \left\langle i^2\right\rangle_{\min} = \left\langle (P_{\mathrm{L}}^{\mathrm{out}})^2\right\rangle \left[1+\left\langle\xi^2\right\rangle\right] \tag{85}$$

Hence we can obtain:

$$\xi_{RMS} \approx \sqrt{\frac{\langle i^2 \rangle_{\min}}{\langle i^2 \rangle_{\max}}} \tag{86}$$

valid in the limit $\sqrt{\langle \xi^2 \rangle} << 1$ rad. This expression allows us to straightforwardly calculate the lock imprecision obtained in the experiment. From the suppression ratio observed in Fig. 2 of the main text near the mechanical resonance frequency, a value of $\langle \xi^2 \rangle = 10^{-6}$ is calculated.

Given knowledge of the imprecision, we can then estimate the magnitude of the decoherence produced. The process ξ can be treated classically, as it typically arises from acoustic and electronic noise in feedback loop used to lock the interferometer. As a result, the fluctuations ξ can in principle be determined. The decoherence rate is then determined by substituting Eq. (21) into Eq. (84), keeping only the first order mechanical fluctuations, from which we find

$$\left\langle (P_{\rm L}^{\rm out})^2 \right\rangle \left\langle \xi^2 \right\rangle = 4\kappa N \lambda^2 \left\langle \xi^2 \right\rangle \left\langle X_{\rm M}^2 \right\rangle \ . \tag{87}$$

We can neglect the correlation term between the light and mechanics, as it is only a small correction in the high-cooperativity regime. The resulting decoherence rate due to lock imprecision can then be written

$$\mu_{\rm lock} = 2\kappa N \lambda^2 \left\langle \xi^2 \right\rangle \ . \tag{88}$$

And then the requirement for the quadratic measurement rate to dominate becomes,

$$\eta \mu_2 > \mu_{\rm lock} \ , \tag{89}$$

or

$$\eta \lambda^2 > \left\langle \xi^2 \right\rangle \ . \tag{90}$$

Given the small magnitude of this decoherence mechanism compared to the term arising from finite detection efficiency, homodyne lock precision is not expected to be a limiting factor in practice.

Mechanical mode mixing: Mechanical mode mixing arises in practice when naturally occurring mechanical modes, other than the one of interest, additionally couple to the optical system. Due to the non-linear nature of the interaction, different mechanical modes will mix with one-another in the measurement signal. Although this effect can occur to any order, we note that higher order interactions will depend on higher powers of the coupling parameter λ, and so provided this parameter is $\ll 1$, it is sufficient to only consider pair-wise interactions. Consequently we begin by considering an interaction Hamiltonian that only includes two mechanical modes

$$H_{\rm int}/\hbar = \sqrt{2} a^\dagger a \left(g X_{\rm M} + g_j X_{{\rm M}(j)} \right) \ . \tag{91}$$

Following the same procedure as earlier, we obtain the input-output relation

$$a_{\rm out} = a_{\rm in} \left(1 - \frac{2\zeta}{1 + i(\lambda X_{\rm M} + \lambda_j X_{{\rm M}(j)})} \right) \ , \tag{92}$$

where we have defined the new optomechanical coupling strengths $\lambda = \sqrt{2} g/(\kappa{+}\gamma)$, and $\lambda_j = \sqrt{2} g_j/(\kappa{+}\gamma)$. Expanding this form in a Taylor series linear in the fundamental mode we obtain

$$a_{\rm out} = a_{\rm in} - \sqrt{2N\kappa} \left(1 - i\lambda X_{\rm M} - 2\lambda\lambda_j X_{\rm M} X_{{\rm M}(j)} + ... \right) \ . \tag{93}$$

The terms linear in the fundamental mode can in principle give rise to a mixing of the state of $X_{\rm M}$. Consider for instance if $X_{{\rm M}(j)}$ is in a position eigenstate, as might be the case if mode j is separately strongly measured. Then, ignoring the outcome of the terms in series Eq. (93) amounts to a trace operation over $X_{{\rm M}(j)}$, which causes mixing of $X_{\rm M}$, or linear position decoherence.

To estimate the magnitude of the decoherence effect of the the mixing terms, we first appreciate that we need only consider the first (quadratic) mixing term. This approximation is valid for low occupation and coupling in modes considered. This term will produce spectral peaks at $\omega_a + \omega_b$ and $\omega_a - \omega_b$. If mode j via hypothetical measurement is forced into a position eigenstate, we can replace the $X_{{\rm M}(j)}$ operator by its measurement outcome. Since the variance of mode j in an initial thermal state is given by $\sigma^2_{X_{{\rm M}(j)}} = \bar{n}_j + 1/2$, on average this measurement outcome will have a

magnitude of $\sqrt{\bar{n}_j + 1/2}$. Then the variance in the mixing term present in the detected amplitude quadrature of the light $X_L = (a + a^\dagger)/\sqrt{2}$ is:

$$\left\langle \left(2\sqrt{N\kappa}\lambda\lambda_j X_{\rm M} X_{{\rm M}(j)}\right)^2 \right\rangle = 4N\kappa \left(\lambda\lambda_j\right)^2 \left(\bar{n}_j + 1/2\right) \left\langle X_M X_M \right\rangle \ . \tag{94}$$

We can compare this expression to the measurement rates obtained in Eq. (22) and identify the effective decoherence rate due to intra-modal mixing as,

$$\mu_{{\rm mix}(j)} = 2N\kappa \left(\lambda\lambda_j\right)^2 \left(\bar{n}_j + 1/2\right) \ . \tag{95}$$

This gives an estimate for the mean decoherence rate produced by mode j. For the quadratic measurement to dominate the decoherence induced by all auxiliary modes of the structure, we require,

$$\eta\mu_2 > \sum_{j=2}^{\infty} \mu_{{\rm mix}(j)} \ , \tag{96}$$

or

$$\frac{\eta\mu_2}{\sum_{j=2}^{\infty} \mu_{{\rm mix}(j)}} > 1 \ . \tag{97}$$

For a string with uniform coupling over its entire length (as is usually the case in electromechanical systems), the relevant product of the square of the coupling parameter λ and the mean occupation $\bar{n}$ for each higher order mode j is related to the fundamental mode coupling rate as

$$\frac{\lambda_j^2(\bar{n}_j + 1/2)}{\lambda^2(\bar{n} + 1/2)} = \frac{1 + (-1)^{j+1}}{2j^3} \frac{\frac{1}{e^{j\epsilon}-1} + \frac{1}{2}}{\frac{1}{e^{\epsilon}-1} + \frac{1}{2}} \ , \tag{98}$$

with the occupation for mode j given by the Bose-Einstein distribution,

$$\bar{n}_j = \frac{1}{e^{j\epsilon} - 1} \ , \tag{99}$$

and $\epsilon = \hbar\omega_{\rm M}/kT$. The assumptions that have been made include uniform mass distribution along the string; a sinusoidal mode-shape function; string eigenfrequencies that are integer multiples of the fundamental and all higher-order modes being in thermal equilibrium at a temperature T. Using this result, Inequality 97 can be evaluated as follows,

$$\begin{aligned} \frac{\eta\mu_2}{\sum_{j=2}^{\infty} \mu_{{\rm mix}(j)}} &= \frac{\eta}{(\bar{n} + 1/2)} \frac{\lambda^2(\bar{n} + 1/2)}{\sum_{j=2}^{\infty} \lambda_j^2(\bar{n}_j + 1/2)} \\ &= \frac{\eta}{(\bar{n} + 1/2)} \left(\sum_{k=1}^{\infty} \frac{\frac{1}{e^{(2k+1)\epsilon}-1} + \frac{1}{2}}{(2k+1)^3(\frac{1}{e^{\epsilon}-1} + \frac{1}{2})} \right)^{-1} \\ &\approx \eta \frac{19.3}{(\bar{n} + 1/2)} > 1 \ , \end{aligned} \tag{100}$$

where the convergent series was numerically approximated at $\epsilon = 19.3$. This implies that such a system, with near-unity detection efficiency, if cooled uniformly to a temperature where the fundamental mode has an occupation less than 19.3 phonons, will not have mode-mixing as a limiting factor for state preparation.

Next we consider a string with a delta-like measurement of mechanical motion at the antinode of the fundamental mode, which while somewhat un-physical, is a useful description of our apparatus in the limit that the string is much longer than the cavity radius. In this case it can be shown that,

$$\frac{\lambda_j^2(\bar{n}_j + 1/2)}{\lambda^2(\bar{n} + 1/2)} = \frac{1 + (-1)^{j+1}}{2j} \frac{\frac{1}{e^{j\epsilon}-1} + \frac{1}{2}}{\frac{1}{e^{\epsilon}-1} + \frac{1}{2}} \ , \tag{101}$$

where same assumptions as before have been used. This form of coupling to the string modes does not lead to convergence in the infinite sum of Inequality 97 at any temperature, and hence it is always unsatisfied. As a result, it can be concluded that delta-like coupling of simple string like-modes precludes quantum state generation. In a more

realistic scenario, where the mechanical string modes interact with the optical mode over a finite length, it is expected the infinite sum of the mixing rates should in fact converge to a finite, albeit large value.

In order to minimise the effect of this decoherence channel, a primary approach should be to use either uniform coupling to string modes, or the use of mechanical oscillators other than simple string structures, such as those discussed in Ref. [8]. Additionally, with application of dilution refrigerator temperatures and side-band cooling, the thermal occupation of the auxiliary modes can be reduced by a factor of 10^5 to 10^6, enabling a quantum regime of state preparation.

Supplementary Note 9: Kraus Operator Approach to Measurements with Optical Loss

In our scheme we utilise a radiation pressure interaction that couples to the mechanical position and not to the mechanical position squared. Hence any optical loss between the interaction and homodyne detection is a loss of mechanical position information and gives rise to decoherence. In this section we extend the Kraus operator approach used in Ref. [5] to incorporate such loss. In general, all types of optical loss and detection inefficiency can be modelled with a single beam-splitter with vacuum on the unused port and tracing over the output 'loss' port, see e.g. Ref. [6]. Quantitatively the conditional mechanical state is

$$\rho_{\rm M}^{\rm out} \propto {\rm Tr}_{\rm V}\left\{{}_{\rm L}\langle X_{\rm L}|\, B e^{i\lambda a^\dagger a X_{\rm M}} \rho\, e^{-i\lambda a^\dagger a X_{\rm M}} B^\dagger |X_{\rm L}\rangle_{\rm L}\right\} , \tag{102}$$

where $\rho = \rho_{\rm M}^{\rm in} \otimes |\alpha\rangle_{\rm L}\langle\alpha| \otimes |0\rangle_{\rm V}\langle 0|$ describes the initial uncorrelated states of the three modes involved, B is the beam-splitter operator defined via $B^\dagger a B = \sqrt{\eta}a - \sqrt{1-\eta}a$, and subscript (V) labels the vacuum loss mode. The conditional mechanical state can also be written as

$$\rho_{\rm M}^{\rm out} \propto \int_{-\infty}^{\infty} {\rm d}P_{\rm V}\, \Upsilon_\eta(P_{\rm V}) \rho_{\rm M}^{\rm in} \Upsilon_\eta^\dagger(P_{\rm V})\, , \tag{103}$$

where

$$\Upsilon_\eta(P_{\rm V}) = {}_{\rm V}\langle P_{\rm V}|\, {}_{\rm L}\langle X_{\rm L}|\, B\, e^{i\lambda a^\dagger a X_{\rm M}}\, |\alpha\rangle_{\rm L}\, |0\rangle_{\rm V}\ . \tag{104}$$

Here, ${}_{\rm V}\langle P_{\rm V}|$ is a phase quadrature projection in the loss port that is averaged over to implement the trace. To second order in $\lambda X_{\rm M}$ this operator is

$$\begin{aligned}\Upsilon_\eta(P_{\rm V}) \simeq & \sqrt{\pi}\ \exp[-\tfrac{1}{2}(\delta X_{\rm L} - \sqrt{2\eta}\alpha\lambda^2 X_{\rm M}^2/2)^2] \\ & \exp[-\tfrac{1}{2}(P_{\rm V} + \sqrt{2(1-\eta)}\alpha\lambda X_{\rm M})^2] \\ & \exp[-i\sqrt{2\eta}\alpha\lambda X_{\rm M}\delta X_{\rm L}] \\ & \exp[i\sqrt{2(1-\eta)}\alpha\lambda^2 X_{\rm M}^2 P_{\rm V}/2] \\ & \exp[i\alpha^2\lambda X_{\rm M}]\, ,\end{aligned} \tag{105}$$

where the amplitude quadrature measurement outcome is $X_{\rm L} = \sqrt{2\eta}\alpha - \delta X_{\rm L}$. The top line is the Bayesian part of the $X_{\rm M}^2$ measurement; the second line describes the decoherence due to the loss of mechanical position information to the environment (recall that $P_{\rm V}$ is 'averaged over' according to Eq. (103)); the third line describes the momentum transfer to the mechanical oscillator from the known optical amplitude quadrature measurement outcome, which can be cancelled with feedback; the fourth line describes the $X_{\rm M}^2$ back-action noise due to the optical loss; and the last line describes the momentum transfer due to the mean photon number, which can also be cancelled with feedback.

Since the measurement outcome $\delta X_{\rm L}$ is known, the optical amplitude noise term may be cancelled via feedback, in the manner discussed in the earlier section. Similarly the mean field α is also known, and the displacement it generates on the mechanics is easily cancelled. Hence the two respectively terms in the Krauss operator may be removed when feed-back is performed, resulting in the following operator

$$\begin{aligned}\Upsilon_\eta(P_{\rm V}) = & \sqrt{\pi}\ \exp[-\tfrac{1}{2}(\delta X_{\rm L} - \sqrt{2\eta}\chi_2 X_{\rm M}^2)^2] \\ & \exp[-\tfrac{1}{2}(P_{\rm V} + \sqrt{2(1-\eta)}\chi_1 X_{\rm M})^2] \\ & \exp[i\tfrac{1}{2}\sqrt{2(1-\eta)}\chi_2 X_{\rm M}^2 P_{\rm V}]\, ,\end{aligned} \tag{106}$$

where the non-dimensional measurement strengths $\chi_1 = \sqrt{N}\lambda$, and $\chi_2 = \sqrt{N}\lambda^2$ have been identified. Further simplification of the measurement operator results by writing the measurement outcomes in terms of estimates of the

mechanical position and position-squared operator outcomes, i.e.

$$\begin{aligned}\Upsilon(Q) =& \sqrt{\pi}\ \exp[-\eta\chi_2^2(X_{\mathrm{M,est}}^2 - X_{\mathrm{M}}^2)^2]\times \\ & \exp[-(1-\eta)\chi_1^2(Q+X_{\mathrm{M}})^2]\times \\ & \exp[-(1-\eta)\chi_1\chi_2 iQX_{\mathrm{M}}^2]\,,\end{aligned} \tag{107}$$

with the new measurement outcome variables $Q = P_{\mathrm{V}}/\sqrt{2}(1-\eta)\chi_1$ and $X_{\mathrm{M,est}}^2 = \delta X_{\mathrm{L}}/\sqrt{2}\eta\chi_2$ describing the estimates of the mechanical position and position squared from the loss port and homodyned-field respectively.

A minimum requirement for this measurement operator to generate non-Gaussian states with Wigner negativity (after performing the trace over Q), is that the linear (Gaussian) measurement strength not exceed the nonlinear (non-Gaussian) measurement strength. That is $\eta\chi_2^2 > (1-\eta)\chi_1^2$, and hence:

$$\lambda^2 > \frac{1-\eta}{\eta}\,, \tag{108}$$

Since the Krauss operator approach can be extended to a continuous time by alternate application of infinitesimal evolution and measurement operators, we expect the non-classicality requirements of the state generated here should remain applicable to the continuous measurement case. In fact comparing this result with Eq. (81) for the case where $\zeta \to 1$, we find the two approaches yield consistent criteria.

Now with an analytical form for the Krauss operator, the precise effect of varying detection efficiency on the non-classicality of the conditional state can be quantified. Our chosen non-classicality criterion is that of Wigner negativity [7]. The negativity of a state (δ) is defined as the following quantity

$$\delta_\rho = \int\int |W_\rho(X,P)|\ dXdP - 1\ , \tag{109}$$

with integrals are implicitly performed between $-\infty$ and $+\infty$ and the Wigner function defined as

$$W_\rho(X,P) = \frac{1}{\pi}\int dY\,\langle X+Y|\,\rho\,|X-Y\rangle\, e^{2iPY}\ . \tag{110}$$

The negativity, δ, may be interpreted as the volume of the negative region of the Wigner function with respect to the ground state volume, and can in principle be arbitrarily large. Using this metric, we characterise the non-classicality of the state produced by the action of the operator (107) on an initial ground state. For a fixed quadratic interaction strength $\eta\chi_2^2$, varying the magnitude of the linear interaction (by appropriately varying the intracavity photon number and coupling strength λ) illustrates the sensitivity of the nonclassicality of the resultant state to loss as shown in Fig. 3. The plot illustrates that Inq. (108) is not a strict bound, and negativity appears even for $\lambda^2\eta/(1-\eta) < 1$.

Figure 4 shows the effect of fixing the intracavity photon number and coupling parameter λ while varying the detection efficiency. Although from Eq. (108) the critical efficiency in this case is 96%, we can see negativity persists even if this criterion is not quite met. Figure 5 illustrates the Wigner function for some of the efficiencies used in the Fig. 4 calculation.

The picture obtained in the Krauss operator picture may be connected to the continuous measurement regime by identifying the connection between the measurement rates, μ_1; μ_2 obtained from the expression for the homodyne photocurrent Eq. (22) with the measurement strengths $\chi_1; \chi_2$ as follows:

$$\mu_2 = 2\kappa N\lambda^4 = 2\kappa\chi_2^2 \tag{111}$$

$$\mu_1 = 2\kappa N\lambda^2 = 2\kappa\chi_1^2 \tag{112}$$

where the equalities follow simply from the definition of the respective quantities. Hence the requirement that $\chi_2 > \chi_1$ for the appearance of negativity in the Wigner function is equivalent the requirement that $\mu_2 > \mu_1$ in the continuous measurement regime. These results motivate our choice of the linear and quadratic measurement rates in the master equation approach in section 10.

Decoherence due to loss of phonon number information to the environment: In the present experiment we utilise the component of the $X_{\mathrm{M}}^2(t)$ signal that oscillates at $2\omega_{\mathrm{M}}$ and do not have access to the DC component due to low frequency technical noise. By writing

$$X_{\mathrm{M}}^2(t) = b^\dagger b + \tfrac{1}{2} + \tfrac{1}{2}(b^2 + b^{\dagger 2})\,. \tag{113}$$

it is seen that $2\omega_{\mathrm{M}}$ component depends on the operator $(b^2 + b^{\dagger 2})$ and the DC term depends on $b^\dagger b$. (The $b^\dagger b$ term is a product of counter-rotating operators and is hence at DC, whereas the b^2 and $b^{\dagger 2}$ are products of co-rotating

field operators and hence gives a signal at $2\omega_{\mathrm{M}}$.) As a consequence, the spectral peaks identified in Eq. (40) may be identified as containing information about the phonon number and the squares of the ladder operators at DC and $2\omega_{\mathrm{M}}$ respectively. In this section we discuss how the loss DC information, and hence phonon number information, to the environment affects quantum states. In particular, we show that this type of decoherence channel does not necessarily eliminate non-classicality in the form of Wigner negativity.

To demonstrate this we use a simple model that couples an initial mechanical state $\rho_{\mathrm{M}}^{\mathrm{in}}$ to an oscillator in its ground state and then we trace over the auxiliary oscillator, i.e.

$$\rho_{\mathrm{M}}^{\mathrm{out}} = \mathrm{Tr}_{\mathrm{V}}\left\{ e^{i\theta b^\dagger b X_{\mathrm{V}}} \rho_{\mathrm{M}}^{\mathrm{in}} \otimes |0\rangle_{\mathrm{V}}\langle 0|\, e^{-i\theta b^\dagger b X_{\mathrm{V}}} \right\} . \tag{114}$$

The trace operation can be reduced to an integral in the Krauss formalism:

$$\rho_{\mathrm{M}}^{\mathrm{out}} \propto \int_{-\infty}^{\infty} \mathrm{d}X\, \Upsilon(X) \rho_{\mathrm{M}}^{\mathrm{in}} \Upsilon^\dagger(X) , \tag{115}$$

where the Krauss operator Υ is given by:

$$\begin{aligned} \Upsilon(X) &= {}_{\mathrm{V}}\langle X|\, e^{i\lambda a^\dagger a X_{\mathrm{V}}}\, |0\rangle_{\mathrm{V}} \\ &= \pi^{-\frac{1}{4}} e^{-\frac{1}{2}X^2} e^{i\lambda a^\dagger a X} . \end{aligned} \tag{116}$$

In Fig. 6 we have plotted the Wigner functions of an initial pure state taken to be $|\psi\rangle \propto |1.7i\rangle + |-1.7i\rangle$ and the states from Eq. (114) for increasing decoherence strength θ. Note that Wigner negativity remains even for very large values of this type of decoherence. Similarly, for an odd superposition state, a negative region is seen to persist in the center of the Wigner function (not shown).

Why this non-classicality remains after losing (complete) quanta number information to the environment can be understood by considering the density matrix in the number basis, i.e. $\rho_{nm} = \langle n|\, \rho\, |m\rangle$. The initial state $|\psi\rangle$ is an even cat state, i.e. the diagonal components ρ_{nn} are populated only when n is even. The decoherence eliminates the off-diagonal components leaving the diagonal components unchanged, thus generating a mixed state. However, ρ_{nn} is not a smooth distribution, and a mixture of even Fock states is still a non-classical state. In light of this observation, we expect that the loss of the DC signal in our experiment, which carries phonon number information, does not preclude the generation of a non-classical state of motion. This will be quantitatively discussed with a master equation approach in 10.

Supplementary Note 10: Continuous Approach using the Master Equation Description

The Krauss operator approach used in the previous section may be thought of as the effect of an instantaneous measurement, or more specifically a measurement that occurs on a timescale much shorter than the time scales associated with the free evolution of the system. The effect of a continuous measurement may be thought of as equivalent to successive application of projective measurements of the form described by the Krauss operator and free evolution of the system given by the hamiltonian propagator. In the limit as the time step associated with each process goes to zero, a description of continuous quantum measurement is obtained. The result being well-known [1, 3], we reproduce it here in the standard stochastic master equation form:

$$d\rho = -\frac{i}{\hbar}[H,\rho]\; dt + \sum_n \left(\mathcal{D}[c_n]\rho\; dt + \mathcal{H}[\sqrt{\eta_n} c_n]\rho\; dW_n\right) \tag{117}$$

where dW is the normally distributed Wiener increment and has expectation value of $\sqrt{dt}$; $\mathcal{D}[c]$ is the Lindblad or collapse superoperator and $\mathcal{H}[c]$ is the measurement superoperator for a particular interaction defined by the operator c. The superoperators are defined in the following way:

$$\begin{aligned} \mathcal{D}[c]\rho &= c\rho c^\dagger - \frac{1}{2}\left(c^\dagger c\rho + \rho c^\dagger c\right) \\ \mathcal{H}[c]\rho &= c\rho + \rho c^\dagger - \left\langle c + c^\dagger \right\rangle \rho \end{aligned} \tag{118}$$

For linear interactions, the Lindblad term tends to cause decay and Gaussian smoothing of the state, whilst the measurement term conditions the state towards an eigenstate of the particular measurement operator, the precise

value of which is determined by the specific trajectory. As a result, the stochastic master equation is then able to describe the evolution of the density matrix considering both measurement and decoherence processes, with the relative strength of each process determined by the magnitude of the detection efficiency, and where, with the correct choice of operators, we need not model the optical sub-system. As further justification that the optical measurement and feedback need not be modelled, the Krauss operator approach of section 9 showed that the effect of measurement and feedback in the optical sub-system can be reduced to a Krauss operator acting only on the mechanical sub-system.

When considering the effect of finite detection efficiency, we are free to choose any type of basis over which to perform the trace in the loss port. For simplicity then, we choose the phase quadrature basis, which gives rise to a pure position measurement (accurate to second order), with a rate of $(1-\eta)\mu_1$. This information, lost to the environment, is treated in the master equation as a linear measurement with zero efficiency. Other decoherence processes, such as mode-mixing, and errors in the homodyne angle, and imperfect feed-back can be accounted for in the master equation simulation by an increase in the linear decoherence rate beyond the limit imposed by finite detection efficiency, as justified in section 8.

The dynamics of the mechanical system under consideration can now be obtained using the master equation, including quadratic measurement of finite efficiency, decoherence due to linear interaction and decoherence due to loss of DC information. The Lindblad terms ($\mathcal{D}[c_n]$) that arise in the master equation are given by the following choice of operators:

Lindblad Process	Rate	Operator (c_n)
Loss	$(1-\eta)\mu_1$	$\sqrt{(1-\eta)\mu_1}(b+b^\dagger)/\sqrt{2}$
X^2 (AC)	$\eta\mu_2$	$\sqrt{\eta\mu_2}(b^\dagger b^\dagger + bb)/2$
X^2 (DC)	$\eta\mu_2$	$\sqrt{\eta\mu_2}(b^\dagger b + bb^\dagger)/2$

We note that due to the leading (linear) term of the OM interaction, a Lindblad process at a rate of μ_1 acts on the mechanics, however due to the action of feedback from the homodyned amplitude quadrature, the decoherence rate is reduced to $(1-\eta)\mu_1$ (as discussed section 7).

The conditioning process ($\mathcal{H}[\sqrt{\eta_n}c_n]$) is given by a single measurement superoperator:

Conditioning Process	Rate	Operator ($\sqrt{\eta_n}c_n$)
X^2 (AC)	$\eta\mu_2$	$\sqrt{\eta\mu_2}(b^\dagger b^\dagger + bb)/2$

This choice of collapse operator is consistent with the feed-back case analysed earlier, and specifically we can see leads to the same coupling rate requirement if the quadratic conditioning rate, $\eta\mu_2$, exceeds the decoherence due to the trace of the linear interaction on the loss port, that is,

$$(1-\eta)\mu_1 < \eta\mu_2 \ , \tag{119}$$

which, consistent with previous results implies,

$$\lambda^2 > \frac{1-\eta}{\eta} \ . \tag{120}$$

The time-dependent solution to the master equation for a particular trajectory can be obtained with direct numerical integration of Eq. (117) using a finite difference method. Here we choose a second order Runge-Kutta integration method (the mid-point method) with 100,000 timesteps per mechanical period, which ensures numerical stability. The density operator is represented as a 50-dimensional matrix.

In addition to the negativity δ, defined previously, it is useful to introduce the mechanical state purity,

$$\gamma_\rho = \mathrm{Tr}[\rho^2] \ , \tag{121}$$

which is equal to 1 for a pure quantum state and 0 for an entirely mixed state.

The simulation in Fig. 7 was then performed with a choice of measurement parameters corresponding to the threshold Inq. (120). The initial state is in thermal equilibrium with the bath with $\bar{n}=1$. Allowing the system to evolve with $T=1/\omega_\mathrm{M}$; $\lambda=0.3$; $Q_M=5000$; $\eta=0.96$; $\mu_1=\omega_M/15\lambda^2$; $\mu_2=\omega_M/15$ from the initial thermal state does produce a coherent superposition state at a finite time that persists for a time on the order of one mechanical cycle. The initial thermal state begins with a purity of $\gamma=0.33$ and a negativity $\delta=0$. As the state evolves under continuous measurement and feed-back, non-classicality is seen to appear in both a finite detection efficiency case (Fig. 7) and naturally also for a case with perfect detection efficiency (Fig. 8). We emphasise that both simulations include the effect of loss of DC information, which has not hindered the generation of highly non-classical states.

Additionally an animation of the state evolution in the finite detection case of Fig. 7, starting at $t = 4.5T$ and ending at $t = 6.4T$, is given as a supplementary animation.

These simulations demonstrate that the method of conditional state preparation outlined here works even in the absence of ground state cooling, and several decoherence processes, namely DC loss and processes that generate effective linear coupling to the environment.

Supplementary Note 11: Mathematical Conventions

Fourier transforms: Define the non-unitary Fourier transform convention:

$$X(\omega) = \int dt\ x(t)e^{i\omega t}; \quad x(t) = \frac{1}{2\pi}\int d\omega\ X(\omega)e^{-i\omega t} \tag{122}$$

Delta Function:

$$\int dt\ \delta(t)e^{i\omega t} = 1; \quad \int dt\ e^{i\omega t} = 2\pi\delta(\omega) \tag{123}$$

Convolution theorem:

$$\begin{aligned} f(t) = g(t)h(t) \implies& \\ F(\omega) =& \frac{1}{2\pi}\int d\omega' G(\omega-\omega')H(\omega') \end{aligned} \tag{124}$$

$$\begin{aligned} F(\omega) = G(\omega)H(\omega) \implies& \\ f(t) =& \int dt' g(t-t')h(t') \end{aligned} \tag{125}$$

Cross-correlation function:

$$C_{xy}(t) = \langle x(0)y(t)\rangle \tag{126}$$

Cross-spectral density in terms of the cross-correlation function:

$$\begin{aligned} S_{xy}(\omega) &= \int dt\ e^{i\omega t}C_{xy}(t) \\ &= \int dt\ e^{i\omega t}\langle x(0)y(t)\rangle \\ &= \int dt\ e^{i\omega t}\int dt'\delta(t')\langle x(t')y(t)\rangle \\ &= \frac{1}{2\pi}\int\int dt\ dt' e^{i\omega t}\int d\omega' e^{i\omega' t'}\langle x(t')y(t)\rangle \\ &= \frac{1}{2\pi}\int d\omega'\ \langle x(\omega')y(\omega)\rangle \end{aligned} \tag{127}$$

For a stationary process, the correlation of x and y is,

$$\begin{aligned} \langle xy\rangle &= C_{xy}(0) \\ &= \int dt\ \delta(t)C_{xy}(t) \\ &= \frac{1}{2\pi}\int dt\int d\omega\ e^{i\omega t}C_{xy}(t) \\ &= \frac{1}{2\pi}\int d\omega\ S_{xy}(\omega) \end{aligned} \tag{128}$$

Correlation functions: The properties of the annihilation operator for the vacuum field are reproduced from Ref. [3]

$$\langle\xi(t)\xi(t')\rangle = 0 \tag{129}$$

$$\left\langle\xi^\dagger(t)\xi^\dagger(t')\right\rangle = 0 \tag{130}$$

$$\left\langle\xi^\dagger(t')\xi(t)\right\rangle = \bar{n}\delta(t-t') \tag{131}$$

$$\left\langle\xi(t)\xi^\dagger(t')\right\rangle = (\bar{n}+1)\delta(t-t') \tag{132}$$

Fourier transforming, we obtain the following relations:

$$\langle \xi(\omega)\xi(\omega')\rangle = 0 \tag{133}$$

$$\left\langle \xi^\dagger(-\omega)\xi^\dagger(-\omega')\right\rangle = 0 \tag{134}$$

$$\left\langle \xi^\dagger(-\omega')\xi(\omega)\right\rangle = 2\pi\bar{n}\delta(\omega+\omega') \tag{135}$$

$$\left\langle \xi(\omega)\xi^\dagger(-\omega')\right\rangle = 2\pi(\bar{n}+1)\delta(\omega+\omega') \tag{136}$$

The mechanical noise operator ξ_P, by contrast is not simply delta-correlated and in fact its correlation function must necessarily be complex to preserve the commutator between X_{M} and P_{M}. Following ref [9] we use the symmetrised form, which is delta-correlated:

$$\begin{aligned}\langle \xi_P(t)\xi_P(t')\rangle_{\mathrm{S}} &= \frac{1}{2}\langle \xi_P(t)\xi_P(t') + \xi_P(t')\xi_P(t)\rangle \\ &= (\bar{n}+\frac{1}{2})\delta(t-t')\end{aligned} \tag{137}$$

Fourier transforming the correlation function gives the corresponding frequency domain relation:

$$\begin{aligned}\langle \xi_P(\omega)\xi_P(\omega')\rangle_{\mathrm{S}} &= \frac{1}{2}\langle \xi_P(\omega)\xi_P(\omega') + \xi_P(\omega')\xi_P(\omega)\rangle \\ &= 2\pi(\bar{n}+1/2)\delta(\omega+\omega')\end{aligned} \tag{138}$$

Supplementary References

[1] Wiseman, H. M., and Milburn, G. J. *Quantum Measurement and Control* (Cambridge University Press, 2010).
[2] Kraus, K. *States, Effects, and Operations* (Springer-Verlag, 1983).
[3] Gardiner, C. and Zoller, P. *Quantum noise: a handbook of Markovian and non-Markovian quantum stochastic methods with applications to quantum optics* (Springer 2004).
[4] Doherty, A. C., Szorkovszky, A., Harris, G. I. and Bowen, W. P. The quantum trajectory approach to quantum feedback control of an oscillator revisited. *Phil. Trans. R. Soc. A* 370, 53385353 (2012).
[5] Vanner, M. R. Selective Linear or Quadratic Optomechanical Coupling via Measurement. *Phys. Rev. X* **1,** 021011 (2011).
[6] Leonhardt, U., and Paul, H. Realistic optical homodyne measurements and quasiprobability distributions. *Phys. Rev. A* **48,** 4598 (1993).
[7] Kenfack, A., and Zyczkowski, K. Negativity of the Wigner function as an indicator of non-classicality. *J. Opt. B* **6,** 396-404 (2004).
[8] Vanner, M. R., Hofer, J., Cole, G. D. & Aspelmeyer, M. Cooling-by-measurement and mechanical state tomography via pulsed optomechanics. *Nat. Commun.* **4,** 724-727 (2013).
[9] Giovannetti, V., and Vitali, D. Phase-noise measurement in a cavity with a movable mirror undergoing quantum Brownian motion. *Phys. Rev. A* **63,** 023812 (2001).
[10] Pirkkalainen, J.-M. et al. Cavity optomechanics mediated by a quantum two-level system. *Nat. Commun.* **6,** 6981 (2015).
[11] Macklin, C. et al. A near quantum-limited Josephson traveling-wave parametric amplifier. *Science* **350,** 307-310 (2015).
[12] Brennecke, F., Ritter, S., Donner, T., & Esslinger, T. Cavity optomechanics with a Bose-Einstein condensate. *Science* **322,** 235–238 (2008).
